# A three-dimensional shear dependent continuum model of platelet aggregation under flow

**DOI:** 10.1371/journal.pcbi.1014241

**Published:** 2026-05-18

**Authors:** David Montgomery, Eric S. Barrientos, Jake M. Grdadolnik, Kelli Hendrickson, Aaron L. Fogelson, Keith B. Neeves, Karin Leiderman

**Affiliations:** 1 Department of Mathematics, University of North Carolina at Chapel Hill, Chapel Hill, North Carolina, United States of America; 2 Department of Biomedical Engineering, University of Colorado Denver | Anschutz, Aurora, Colorado, United States of America; 3 Departments of Mathematics and Biomedical Engineering, University of Utah, Salt Lake City, Utah, United States of America; 4 Department of Pediatrics, Hemophilia and Thrombosis Center, University of Colorado Anschutz, Aurora, Colorado, United States of America; 5 Department of Biochemistry & Biophysics, University of North Carolina at Chapel Hill, ‌‌Chapel Hill, North Carolina, United States of America; 6 Computational Medicine, University of North Carolina at Chapel Hill, Chapel Hill, North Carolina,‌‌ United States of America; University of York, UNITED KINGDOM OF GREAT BRITAIN AND NORTHERN IRELAND

## Abstract

Platelet aggregation under flow is a key component of hemostasis, strongly influenced by shear-dependent interactions mediated by von Willebrand factor (vWF). We present a three-dimensional continuum model that incorporates shear-dependent platelet adhesion, cohesion, and activation. The model tracks seven platelet species and integrates shear-dependent kinetics for vWF-mediated binding and activation. Parameterization was guided by microfluidic experiments under controlled shear rates (300/s and 1500/s) with platelet activation pathways inhibited. Simulations reproduce experimental aggregate growth and occlusion dynamics in both straight channels and physiologically relevant extravascular geometries, where shear rates exceed 8000/s. Functional forms for shear-dependent on- and off-rates were implemented using piecewise and nonlinear scaling: on-rates exhibit double-threshold behavior with saturation at high shear, while off-rates combine linear and exponential terms to capture bond lifetime changes under extreme shear. Simulations using these rate forms reproduced occlusion times within the experimentally observed range. Qualitative comparisons with microfluidic imaging further demonstrated that the model reproduces intrathrombus heterogeneity, including the core–shell architecture with activated platelets concentrated near the collagen surface and unactivated platelets forming an outer shell. These results provide mechanistic insight into how shear-dependent vWF-mediated interactions regulate thrombus growth and occlusion. By linking microfluidic data with continuum-scale modeling, this framework provides a computationally efficient platform to study shear-regulated platelet aggregation and its contribution to hemostatic occlusion under physiologic and pathologic flow conditions.

## Author summary

When a blood vessel is injured, platelets aggregate to form a clot. A key player in this process is von Willebrand factor (vWF), which unravels at high shear rates to help platelets adhere and aggregate. While several computational models have simulated platelet aggregation, few capture these shear-dependent interactions in three dimensions and on physiological timescales. Here, we developed a three-dimensional computational model that focuses on platelet aggregation and its dependence on shear. Using microfluidic experiments, we measured platelet aggregation at different shear rates and used those data to calibrate the model. Our simulations closely matched experimental results and revealed how shear-dependent bond lifetimes influence clot growth and vessel occlusion times. The model reproduces occlusion times within experimentally observed ranges and enables simulation of shear conditions that exceed those measurable in vitro. This work offers a computationally efficient way to study clot formation under high-shear conditions on minute timescales, providing new insights into the physical mechanisms that regulate hemostasis.

## Introduction

Normal blood clotting (hemostasis) consists of two tightly coupled components: a biomechanical process of platelet aggregation and a biochemical process of coagulation. Platelets first form transient bonds between their GPIb receptors and von Willebrand factor (vWF) adsorbed to collagen in the vessel wall. Additional bonds form between GPVI receptors and collagen, which signal platelets to secrete granule contents and activate their transmembrane integrins. Activated integrins enable firm adhesion and platelet–platelet cohesion: $\alpha_{\text{IIb}}\beta_{3}$ binds fibrin(ogen) to bridge platelets, while $\alpha_{2}\beta_{1}$ reinforces adhesion to collagen. Platelets bound to vWF and collagen can be further activated by soluble agonists such as thrombin, ADP, and thromboxane A_2_ (TXA_2_), leading to robust and sustained $\alpha_{\text{IIb}}\beta_{3}$ activation for fibrin(ogen) binding. Additional platelets can join the growing aggregate through vWF-mediated cohesion, whereby a mobile platelet binds to vWF bound to an already adherent platelet via GPIb.

**Fig 1:**
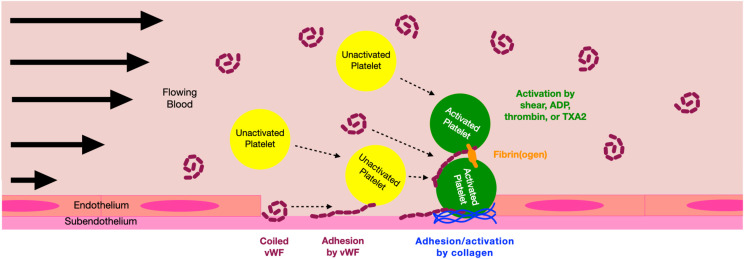
vWF-mediated platelet aggregation. Upon vascular injury, vWF present in the subendothelium uncoils due to shear stress and binds transiently to a platelet’s GPIb receptor, effectively decelerating the platelet near the injury. The platelet subsequently adheres to subendothelial-bound collagen through its GPVI receptor. vWF in the plasma then binds to the subendothelial-bound platelet, enabling cohesion between bound and fluid-phase platelets. After vWF-mediated cohesion occurs, the recently bound platelet can be activated through shear stress, ADP, thrombin, or thromboxane, stimulating the $\alpha_{\text{IIb}}\beta_{3}$ integrin and enabling binding through fibrinogen.

In recent years, particular attention has been paid to the role of vWF in mediating platelet aggregation under high shear [1–4]. The unique structure of vWF enables it to respond to a variety of blood flow conditions, transitioning from a compact, coiled conformation at low shear rates [5,6] to an extended, threadlike state at high shear [7,8]. In this elongated form, binding sites on vWF become exposed, facilitating interactions with platelets and subendothelial collagen [9]. However, the vWF–GPIb bond exhibits rapid dissociation, producing transient adhesion that causes platelets to roll along the vessel wall and prolong their contact time. This temporary interaction promotes platelet activation through GPVI receptors and firm adhesion via integrin-mediated binding. See Fig 1 for a schematic of this vWF-dependent aggregation.

At the same time that platelet aggregation is occurring, coagulation is initiated when plasma clotting factor (F)VIIa binds to tissue factor at the injured vessel wall. Small amounts of thrombin are generated on adherent, collagen-activated platelets and transported by diffusion and flow to activate nearby platelets, which then become procoagulant by exposing negatively charged phosphatidylserine (PS) on their membranes [10,11]. This PS exposure provides a catalytic surface for thrombin-mediated activation of additional coagulation factors (FV, FVIII, FXI), amplifying thrombin generation to levels sufficient for fibrin formation and clot stabilization [10]. Together, these biomechanical and biochemical processes create a dynamic environment where platelet adhesion, cohesion, and coagulation interact under varying shear conditions.

Computational models of hemostasis have provided important biological insights into the coupled roles of flow, platelet aggregation, and coagulation. Across a range of modeling frameworks, these studies have shown how transport and hemodynamics regulate thrombus growth, how platelet–platelet and platelet–wall interactions contribute to aggregate formation, and how clot structure influences stability and embolization. Prior work has also established the importance of shear-dependent platelet adhesion, biochemical amplification pathways in thrombin generation, and the role of clot microstructure in permeability, deformation, and flow redistribution. Collectively, these approaches have advanced understanding of clot formation under physiological and pathological conditions, while revealing trade-offs among mechanistic detail, computational cost, and accessible timescales. However, there remains a lack of computational frameworks that simultaneously capture shear-dependent platelet aggregation, platelet surface coagulation reactions, evolving thrombus structure, and physiologic timescales within a three-dimensional, computationally tractable model.

Many computational models have focused on cell and fluid mechanics [12–22], predicting clot initiation, platelet aggregation, and flow-mediated transport in preformed clots and complex geometries, with less emphasis on incorporating coagulation processes. Recent studies have further leveraged image-based and experimentally informed clot geometries, as well as continuum models of flow and transport within established thrombi, to investigate how thrombus microstructure influences local flow, transport, and stress distributions under varying shear [23–25]. These approaches provide detailed insight into clot–flow interactions, but often treat clot structure as prescribed rather than evolving, or they evolve over limited timescales.

Some computational models have addressed both platelet mechanics and coagulation. These approaches have provided insight into platelet deformation, cell–cell interactions, and the coupling between reaction–diffusion processes and fluid flow at the cellular scale. Particle-based approaches such as dissipative particle dynamics (DPD) or transport (t)DPD simulate platelet and/or red blood cell membrane deformation along with reaction-diffusion processes. These methods are computationally expensive and in many cases require accelerated kinetics to remain tractable when resolving large numbers of particles per cell [17,26–28]. However, these approaches excel at resolving microscale structure and mechanics, including membrane deformation and near-surface transport, providing insight into platelet and red blood cell behavior that complements continuum-scale models. Simplified DPD models representing platelets as single particles improve efficiency, but have only been developed for 2D [29–31]. Notably, such approaches have demonstrated the ability to link receptor-level adhesion kinetics to emergent thrombus structure, providing insight into how microscale interactions give rise to spatial organization such as core–shell architectures. Other multiscale frameworks, such as lattice-based cellular Potts models, incorporate individual platelets but simulate‌‌ coagulation via domain-averaged ODEs, which lead to thrombin generation predictions that differ from experimental observations [32–37]. Nevertheless, these models provide an important framework for coupling discrete platelet dynamics with continuum blood flow, capturing emergent thrombus structure and flow–thrombus interactions. Lattice-based kinetic Monte-Carlo models of platelet aggregation, activation, and internal signaling have also been developed, with and without vessel-wall-restricted coagulation [38–41], but these typically represent the clot as a solid with an evolving boundary, without explicitly resolving porosity and internal flow–coagulation coupling. Despite this, these models provide a powerful multiscale framework that integrates stochastic platelet dynamics with intracellular signaling and flow, and have recently been extended to incorporate donor-specific platelet phenotypes [42], enabling patient-specific prediction of thrombus growth and drug response.

Our prior work introduced platelet-surface-dependent coagulation reactions within a continuum framework, without incorporating mechanical platelet aggregation or shear-dependent aggregation [43–45]. Although these reactions are not included in the present study, this capability is already embedded within the clotFoam framework and can be activated with a simple code flag. This flexibility motivates our use of a continuum formulation, as it enables integration of shear-dependent platelet aggregation with platelet-surface coagulation reactions within a unified modeling framework.

Related continuum modeling approaches have also examined flow-mediated platelet aggregation and occlusion dynamics. For example, Link et al. [46] developed a model of extravascular clot formation that captures platelet adhesion, cohesion, and activation under flow, demonstrating how shear-dependent transport and agonist dilution influence aggregate growth and occlusion. These results highlight the importance of coupling platelet aggregation with flow-mediated transport processes, consistent with the framework used in the present study.

Complementary studies have examined clot deformation, permeability, and embolization under flow, demonstrating how clot microstructure and viscoelastic properties influence stability, transport, and detachment [47–49]. These models typically focus on preformed or partially formed clots and do not explicitly resolve the coupled processes of platelet adhesion, aggregation, and thrombus growth from initiation. However, they provide important insight into clot mechanics and failure, particularly in predicting deformation and fracture under applied flow conditions.

Relative to vWF modeling, Du et al [21,50] presented a two-phase continuum model of platelet aggregation that tracked the concentrations of two types of platelet-platelet bonds, ones comprised of fibrinogen bound to platelet integrin $\alpha_{\text{IIb}}\beta_{3}$ receptors, and ones comprised of vWF bound to platelet GPIb receptors. The integrin receptors were in a high-affinity state only for activated platelets; the GPIb receptors were constitutively available (even on unactivated platelets) and vWF’s ability to bind to them increased substantially with sufficiently high shear rate to reflect stretching of vWF molecules at high shear. Molecular scale experimental data about bond formation and breaking were used to estimate model parameters. That model also included an activation signal transduced by vWF-GPIb bonds. Patel [51] developed a non-spatial model of platelet aggregation which included the dynamics of vWF stretching as well as the dynamics of vWF- and fibrinogen-mediated bond formation and breaking. By explicitly tracking the concentrations of bonds, these models were able to consider fewer platelet states than we do in this paper. Zhussupbekov et al [3] also describes a two-phase continuum model of platelet aggregation that included vWF dynamics and bond formation, enabling representation of platelet–flow interactions within a continuum framework.

Other studies have incorporated shear effects into platelet adhesion modeling. Govindarajan et al. expanded on our previous work [43] and added shear-dependence to the adhesion on and off rates and assumed linear dependence on wall shear rate; Yazdani et al. introduced shear dependence in a discrete Morse potential approach to platelet binding [28]; Shankar et al. proposed a shear-rate-dependent scaling law exhibiting double-threshold behavior, with a maximum scaling reached around 8000/s [40], where vWF adopts its most extended conformation. Collectively, these studies highlight the critical role of shear in regulating vWF-mediated platelet interactions.

In this study, we focus exclusively on platelet aggregation to isolate and characterize the shear-dependent adhesion and cohesion mechanisms mediated by vWF. To isolate mechanical contributions, our study omits the coagulation reactions and builds on our previous four-species continuum framework [43–45], incorporating shear-dependent activation, adhesion, and cohesion processes for platelets. These features, absent from earlier models, are essential for capturing the influence of shear on platelet aggregation. The model is calibrated with one experimental model of platelet adhesion and cohesion assays, and validated by another with a different geometry and flow conditions. This continuum-based approach maintains computational efficiency, enabling simulation over physiologic timescales while capturing platelet surface interactions critical to clot growth. This framework provides a foundation for integrating shear-dependent platelet aggregation with platelet-surface coagulation reactions in future studies. In this study, we focus exclusively on platelet aggregation to isolate and characterize the shear-dependent adhesion and cohesion mechanisms mediated by vWF. To isolate mechanical contributions, our study omits the coagulation reactions and builds on our previous four-species continuum framework [43–45], incorporating shear-dependent activation, adhesion, and cohesion processes for platelets. These features, absent from earlier models, are essential for capturing the influence of shear on platelet aggregation. The model is calibrated with one experimental model of platelet adhesion and cohesion assays, and validated by another with a different geometry and flow conditions. This continuum-based approach maintains computational efficiency, enabling simulation over physiologic timescales while capturing platelet surface interactions critical to clot growth. This framework provides a foundation for integrating shear-dependent platelet aggregation with platelet-surface coagulation reactions in future studies.

## Materials and methods

### Ethics statement

The study received Institutional Review Board approval from the Colorado Multiple Institutional Review Board in accordance with the Declaration of Helsinki (COMIRB #09–0816). Written informed consent was obtained from all participants prior to participation, using the COMIRB-approved informed consent process.

### Mathematical model

The model developed in this study is a direct extension of our previous spatio-temporal model of platelet aggregation and coagulation under flow [43]. That model included continuum descriptions of platelets that were mobile or bound, activated or unactivated, within a dynamic fluid environment, along with solute advection, diffusion, and reaction. In that framework, bound platelets adhered solely through constant binding rates and local platelet concentrations, with no dependence on shear rate. The governing equations for the new shear-dependent platelet aggregation model are also represented with continuum descriptions consistent with our previous work [43,45,52]. Blood is modeled as an incompressible Newtonian fluid governed by the incompressible Navier–Stokes–Brinkman equations:


$$\begin{array}{c} \rho \frac{\partial \vec{𝐮}}{\partial t}+\rho (\vec{𝐮}\cdot \nabla )\vec{𝐮}=-\nabla p+\mu \nabla^{2}\vec{𝐮}-\mu \alpha (\theta^{B})\vec{𝐮}, \end{array}$$ (1)

$$\begin{array}{c} \nabla \cdot \overset{\to }{u}=0. \end{array}$$ (2)

$\vec{𝐮}(\vec{𝐱},t)$ is the fluid velocity, $p(\vec{𝐱},t)$ is pressure, ρ is the fluid density, and μ is the dynamic viscosity.

The last term, $-\mu \alpha (\theta^{B})\vec{𝐮}$, represents a frictional resistance to the fluid arising from the presence of a growing platelet aggregate, $\theta^{B}$. The number fraction, $\theta^{B}$, is the ratio of the sum of all bound platelets at a spatial location to the maximum packing density of platelets, *P*_max_. The resistance coefficient, $\alpha (\theta^{B})$, increases with thrombus density, reflecting reduced permeability of the aggregate. This dependence is modeled using a Carman–Kozeny-type relation,


$$\begin{array}{c} \alpha (\theta^{B})=C_{CK}(0.6\theta^{B}{)}^{2}/(1-0.6\theta^{B}{)}^{3}, \end{array}$$ (3)

where *C*_*CK*_ = 10^6^ mm^-2^.

The model incorporates the role of vWF in platelet adhesion, cohesion, and activation through the definition of seven distinct platelet species, each represented as a number density (plts/mm^3^). These species enable tracking of platelets that are (i) reversibly bound to vWF via GPIb receptors, (ii) irreversibly bound through fibrin(ogen)-mediated activation of the $\alpha_{\text{IIb}}\beta_{3}$ integrin, and (iii) activated by subendothelial collagen via the GPVI receptor. The seven platelet species are defined as follows:


$$\begin{array}{c} \begin{array}{c} P^{m,u}:\text{mobile, unactivated},\\ P^{m,a}:\text{mobile, aggregatory},\\ P^{bv,u}:\text{bound via vWF, unactivated (unable to bind via fibrinogen)},\\ P^{bv,a}:\text{bound via vWF, aggregatory and secretory},\\ P^{bf,a}:\text{bound via fibrin(ogen), aggregatory and secretory},\\ P^{se,u}:\text{subendothelial-bound via vWF, unactivated},\\ P^{se,a}:\text{subendothelial-bound via collagen, aggregatory and secretory. } \end{array} \end{array}$$

**Fig 2:**
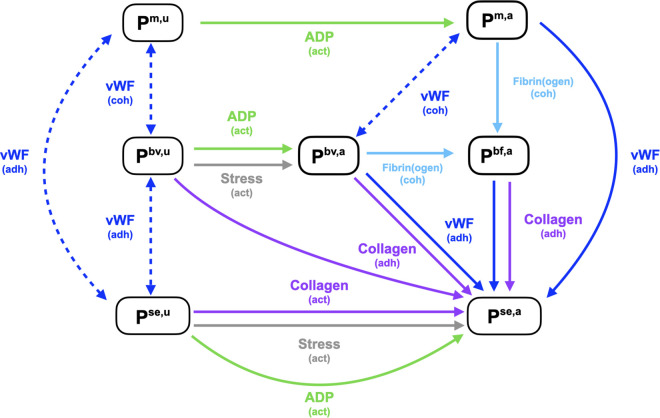
Schematic of platelet model. Transition with dotted lines represent transient binding via vWF, while solid lines depict irreversible state changes. Arrows show the direction of the state change.

A schematic of the shear dependent platelet aggregation model is provided in Fig 2. The full list of reactions and partial differential equations are included in S1 Appendix. The platelet model can described in terms of the distinct pathways originating from the naturally occurring platelets *P*^*m*,*u*^ as summarized below:

1. When a vessel is injured, *P*^*m*,*u*^ platelets adhere directly to the subendothelium via vWF, and transition to *P*^*se*,*u*^.
2. *P*^*se*,*u*^ platelets are activated by collagen, shear stress or ADP, transitioning to *P*^*se*,*a*^. At this stage, they begin secreting ADP to recruit additional platelets for adhesion and cohesion at the injury site.
3. *P*^*m*,*u*^ platelets also cohere to other bound platelets through vWF binding to GPIb receptors, transitioning to *P*^*bv*,*u*^.
4. If *P*^*bv*,*u*^ platelets contact the subendothelium, they bind directly to the subendothelium via vWF through GPIb receptors, transitioning to *P*^*se*,*u*^. Activation by collagen transitions the platelet to *P*^*se*,*a*^.
5. Alternatively, *P*^*bv*,*u*^ platelets can be activated by shear via stress on vWF bonds through GPIb, or by agonists such as ADP or thrombin, transitioning to *P*^*bv*,*a*^.
6. *P*^*bv*,*a*^ platelets, having already been stimulated by agonists, express high-affinity $\alpha_{\text{IIb}}\beta_{3}$ integrin receptors and can irreversibly aggregate with other activated platelets via fibrin(ogen) binding, transitioning to the *P*^*bf*,*a*^ state.
7. As ADP and thrombin generation increases, *P*^*m*,*u*^ platelets can be activated by these agonists to become *P*^*m*,*a*^, providing an additional pathway for irreversible binding through fibrin(ogen).
8. *P*^*m*,*a*^ platelets can also adhere and aggregate via vWF binding, becoming *P*^*bv*,*a*^ or *P*^*se*,*a*^.

It is assumed that *P*^*m*,*a*^ platelets secrete ADP only after becoming bound, and not before. This assumption is based on the time it takes for a platelet to pass over the adhesion region, which is a fraction of the total secretion time. Binding via vWF is always reversible, except for the mobile activated platelets, where we assume irreversible binding to the subendothelium via vWF. All binding via fibrinogen and collagen is assumed to be irreversible. A defining characteristic of the model is the ability for the unactivated platelets to aggregate and embolize via vWF-mediated binding.

Shear-dependence is incorporated into the model by varying the kinetic rates associated with vWF-mediated adhesion, cohesion and activation based on the local shear rate, $\dot{\gamma }$ (1/s). The shear rate is calculated as:


$$\begin{array}{c} \dot{\gamma }=\sqrt{2D:D}, \end{array}$$ (4)

where the“:” denotes a double inner product of two second rank tensors, and **D** is the symmetric part of the velocity gradient tensor:


$$\begin{array}{c} D=\frac{1}{2}(\nabla \vec{𝐮}+(\nabla \vec{𝐮}{)}^{T}). \end{array}$$ (5)

To demonstrate the form of the governing equations for each platelet species, Eq (6) describes the evolution of the mobile unactivated platelets. The full seven-species model is included in S1 Appendix and model parameters are defined in S2 Appendix.


$$\begin{array}{c} \begin{array}{cc} \frac{\partial P^{m,u}}{\partial t}=\;\;\; & -\underset{\text{Transport via advection and diffusion}}{\underbrace{\nabla \cdot \{W(\theta^{T})(\vec{𝐮}P^{m,u}-D_{P}\nabla P^{m,u})\}}}\\ & -\underset{\text{Adhesion to subendothelium via vWF}}{\underbrace{k_{{\text{adh}}^{+}}^{\text{vWF}}(\dot{\gamma })H_{\text{adh}}(\vec{𝐱})P_{\text{max}}(1-\theta^{B})P^{m,u}}}\;\;\;\;\;+\;\;\underset{\text{Unbinding due to shear}}{\underbrace{k_{{\text{adh}}^{-}}^{\text{vWF}}(\dot{\gamma })P^{se,u}}}\\ & -\underset{\text{Cohesion with bound species via vWF}}{\underbrace{k_{{\text{coh}}^{+}}^{\text{vWF}}(\dot{\gamma })[g(\eta^{U})+g(\eta^{A})]P_{\text{max}}P^{m,u}}}\;\;+\;\;\underset{\text{Unbinding due to shear}}{\underbrace{k_{{\text{coh}}^{-}}^{\text{vWF}}(\dot{\gamma })P^{bv,u}}}\\ & -\underset{\text{Activation by ADP}}{\underbrace{A_{ADP}([ADP])P^{m,u}}}. \end{array} \end{array}$$ (6)

Here, the first term accounts for the hindered transport of the mobile unactivated platelets, where the advective and diffusive fluxes are scaled by a hindered transport function developed in our prior work, [43]


$$\begin{array}{c} W(\theta^{T})=\tanh(\pi (1-\theta^{T})), \end{array}$$ (7)

that depends on the total platelet fraction $\theta^{T}$, which is the ratio of the sum of all platelet species to the maximum packing density *P*_max_. To account for the size of the platelets, their transport is hindered in the regions where there are high number densities of platelets due to the thrombus of bound platelets.

The second and third terms represent shear-dependent platelet adhesion to, and detachment from, vWF exposed on the subendothelium. In our previous work [45], the adhesion function $H_{\text{adh}}(\vec{𝐱})$ was defined as unity for finite-volume cells located within one platelet diameter of the subendothelium, and zero elsewhere. In the present study, this region is instead defined using a quasi-random sampling method, as detailed and evaluated in S3 Appendix, where we assess its impact on adhesion site distribution and thrombus formation. The fourth and fifth terms model shear-dependent cohesion between, and detachment from, previously bound platelets, which similar to our previous work [44], is scaled by a binding affinity function g(η). The final term captures platelet activation mediated by the agonist ADP.

The binding affinity function in the vWF-mediated cohesion term is:


$$\begin{array}{c} g(\eta )=\frac{g_{0}(\eta -\eta_{t}{)}^{3}}{\eta_{*}^{3}+(\eta -\eta_{t})}, \end{array}$$ (8)

where $\eta_{t}$ is a threshold value for which there is no binding, $\eta_{*}+\eta_{t}$ is the value of η at which g(η) changes the most rapidly, and $g_{0}=\frac{\eta_{*}^{3}+(1-\eta_{t})}{(1-\eta_{t}{)}^{3}}$ so that *g*(1) = 1. Previously, the binding affinity function was dependent on a single nondimensional virtual substance η, which diffused the bound platelet fraction $\theta^{B}$ a specified distance in order to account for platelet size in platelet-platelet cohesion [45]. There, η was calculated using a single step of a diffusion solver with an initial value of $\eta_{0}=\theta_{n}^{B}$ [45]. Here we developed two distinct types of cohesion: (i) vWF-mediated cohesion and (ii) fibrinogen-mediated cohesion. To prevent double counting in the equations for *P*^*m*,*u*^ and *P*^*m*,*a*^, where cohesion can occur through both vWF and fibrinogen, we defined two virtual substances, denoted by $\eta^{U}$ and $\eta^{A}$. These correspond to unactivated and activated bound platelet populations and depend on their respective platelet fractions, $\theta^{U}$ and $\theta^{A}$, respectively. The virtual substances are calculated using a diffusion process [45]. The diffusion coefficients used for platelet species and virtual substances are consistent with those used in [45] and are summarized in Supplementary Section S2. The initial values in each iteration are set to:


$$\begin{array}{c} \eta_{0}^{U}=\frac{P^{bv,u}(\vec{𝐱},t_{n})+P^{se,u}(\vec{𝐱},t_{n})}{P_{\text{max}}}=\theta_{n}^{U}, \end{array}$$ (9)

$$\begin{array}{c} \eta_{0}^{A}=\frac{P^{bv,a}(\vec{𝐱},t_{n})+P^{bf,a}(\vec{𝐱},t_{n})+P^{se,a}(\vec{𝐱},t_{n})}{P_{\text{max}}}=\theta_{n}^{A}. \end{array}$$ (10)

For all simulations conducted in this paper, we assumed that the platelets coming into the computational domain were spatially distributed to reflect platelet margination within the rectangular channel. Details of this derivation of the spatial profiles are in S4 Appendix.

### Numerical methods

The governing equations are solved using the open-source software package *clotFoam*, develep by our group and built on the OpenFOAM-v9 transient solver framework [45]. *clotFoam* utilizes a cell-centered finite volume method for spatial discretization and Crank-Nicholson for temporal discretization. The fluid solver (1)-(2) implementation is the predictor-corrector PISO algorithm that solves the momentum equation once per timestep with pressure and velocity corrections. The Brinkman term in (3) is treated implicitly as a source term to ensure that the pressure corrections are influenced by the presence of the porous media.

*clotFoam* transports platelet and biochemical species (e.g., (6) and S1 Appendix) through the advection, diffusion reaction (ADR) equations. The software incorporates fractional step method to decouple the transport from the reaction terms at a fixed number of sub steps, which are solved using a fourth-order Runge-Kutta method. For all cases considered, the reaction sub-step timestep is half that of the advection and diffusion timestep. In the present study, the reduced coagulation model present in *clotFoam* is disabled to focus exclusively on the mechanisms of shear-dependent platelet aggregation. Details of the numerical discretization, computational resolution, and hardware used for simulations are provided in S5 Appendix.

### Parameter estimation and calibration

The platelet model includes eight kinetic parameters describing adhesion ($k_{{\text{adh}}^{+}}^{\text{vWF}}$, $k_{{\text{adh}}^{-}}^{\text{vWF}}$, $k_{\text{adh}}^{\mathrm{col}}$), cohesion ($k_{{\text{coh}}^{+}}^{\text{vWF}}$, $k_{{\text{coh}}^{-}}^{\text{vWF}}$, $k_{\text{coh}}^{\mathrm{fbg}}$), and activation ($k_{\text{act}}^{\mathrm{vWF}}$, $k_{\text{act}}^{\mathrm{col}}$). Five of these govern vWF-mediated adhesion, cohesion, and activation and are treated as shear-dependent, while the remaining parameters describe collagen-induced adhesion and activation at the subendothelial surface and fibrin(ogen)-mediated cohesion.

**Fig 3:**
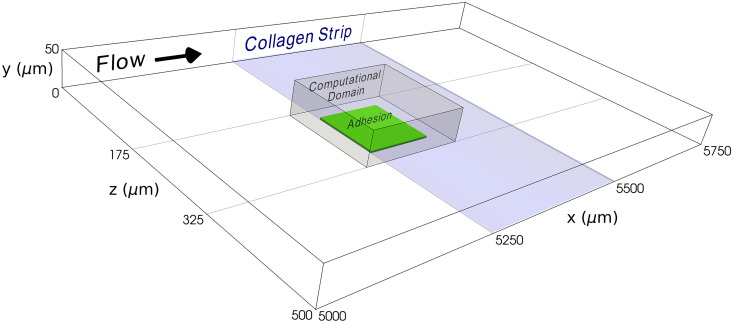
The computational domain. The microfluidic device has dimensions (ℓ,w,h)= (8000, 500, 50) μm. Blood is perfused over a 250×500μm^2^ collagen strip. The leading edge of the collagen strip is located approximately 5250 μm from the inlet of the microfluidic device. For the sake of computational efficiency, the computational domain represents a fraction of the microfluidic device with dimensions (ℓ,w,h)= (160, 150, 50) μm. The computational adhesion region is centered in the computational domain with‌‌ dimensions 100 × 100 μm^2^.

Model calibration was guided by microfluidic experiments performed under thrombin-inhibited conditions at shear rates of 300/s and 1500/s. In these experiments, ADP and TXA_2_ were inhibited to isolate shear-dependent platelet mechanisms. To reduce computational cost, initial exploration of the parameter space was performed using 2D simulations. Candidate parameter sets were then evaluated and refined using 3D simulations in the domain shown in Fig 3. Because clot formation is inherently 3D, parameters identified from 2D simulations were not assumed to be directly transferable to 3D, but instead served to constrain the parameter search space.

Parameters were estimated using a two-stage grid search. A coarse search spanning approximately ±2 orders of magnitude around nominal values from prior work or literature was first used to identify feasible regions of parameter space, followed by a refined search within ±1 order of magnitude using multiplicative steps of 2–5. Parameters were tuned sequentially by minimizing the sum of squared error over the full time course.

Using data at 300/s, we estimated rates governing vWF-mediated adhesion (via GPIb), cohesion (via GPIb and $\alpha_{\text{IIb}}\beta_{3}$), and activation (via vWF and collagen). The collagen adhesion rate was adapted from prior work [43,53], and ADP-related parameters were carried over from previous models [43,45], with minor modifications to prevent spurious activation in regions of low platelet density due to the continuum formulation. Shear-dependent parameters were then refined using data at 1500/s while holding all other parameters fixed. A functional form for vWF-mediated activation was adapted from prior work [3], and parameter values at low and high shear were linearly interpolated to define continuous shear-dependent rates.

Because the model is nonlinear, multiple parameter combinations can produce similar aggregate behavior. Accordingly, parameter ranges were constrained based on prior studies, and calibration was performed to reproduce experimental observations across multiple conditions. Only parameters associated with shear-dependent adhesion and cohesion were varied, while the remainder of the model structure and parameters were retained from previously validated frameworks [43,45,52]. All parameter values used in the simulations are provided in S2 Appendix.

### Functional forms for shear-dependent on- and off-rates

We evaluated multiple candidate functional forms and selected those that best reproduced experimental occlusion times. For all shear-dependent functions, we assumed a linear form up to 2000/s shear, based on calibrations in the straight channel microfluidic channel. Beyond 2000/s, alternative extensions of $k_{+}(\dot{\gamma })$ and $k_{-}(\dot{\gamma })$ were explored to capture shear-dependent platelet interactions at higher shear rates: piecewise-linear and nonlinear.

**Fig 4:**
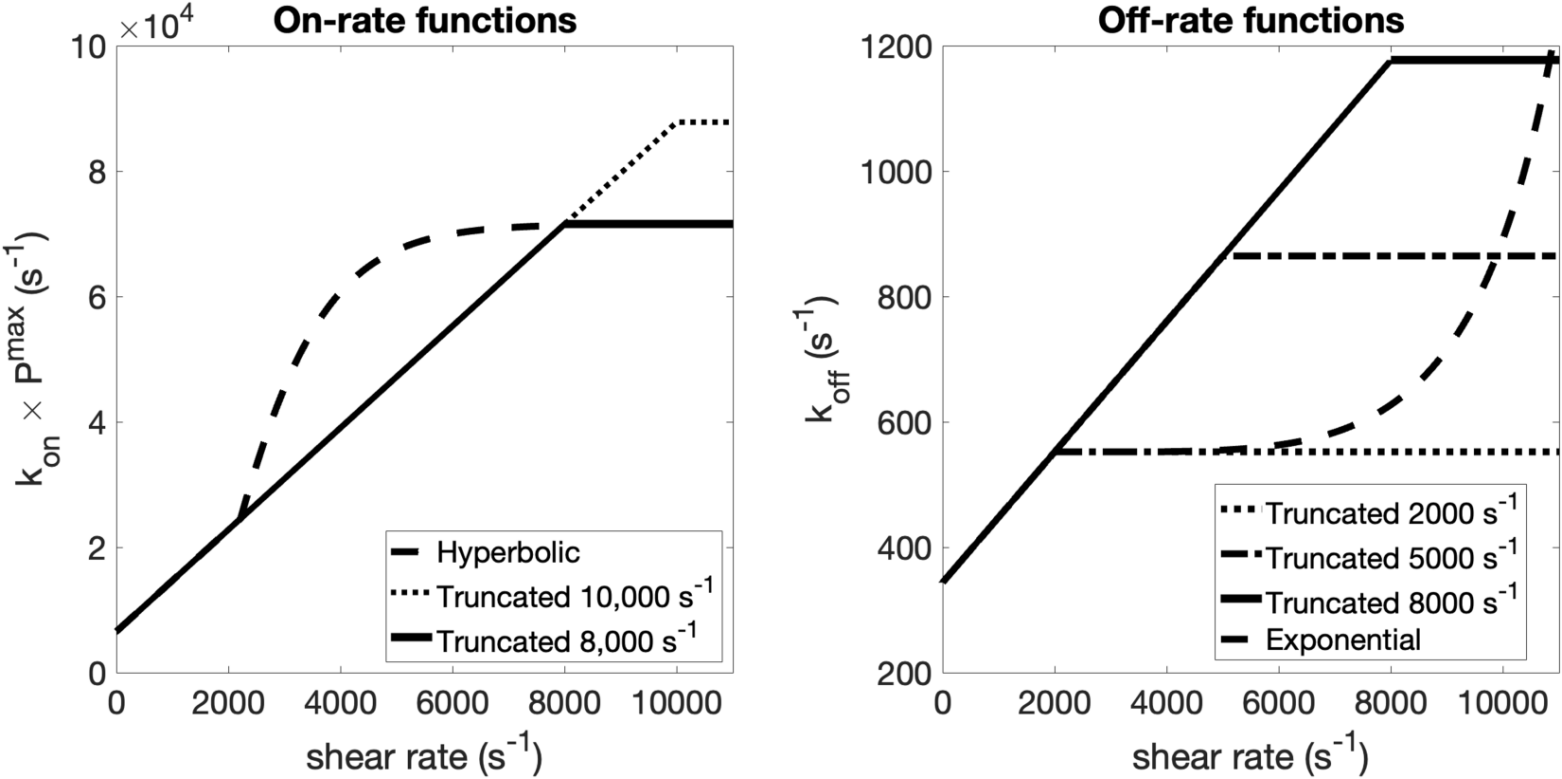
Shear-rate dependent functional forms of adhesion and cohesion. Functional forms for the on-rates $k_{+}(\dot{\gamma })$ (left) and off-rates $k_{-}(\dot{\gamma })$ (right) used to extend shear dependence beyond 2000/s. Piecewise-linear functions were tested for both on- and off-rates. Nonlinear forms include a hyperbolic tangent function for the on-rate, representing a faster-than-linear increase for intermediate shear rates, and an exponential relationship for the off-rate, reflecting reduced bond lifetimes as shear increases.

We first considered truncating the linear forms at selected shear rates, resulting in piecewise-linear functions. Building on this, we introduced functional forms consisting of linear scaling up to a 2000/s, followed by a smooth, non-linear transition to higher shear rates. For $k_{+}(\dot{\gamma })$, we assumed two, piecewise-linear forms with truncations at 8000/s and 10000/s, an one using a hyperbolic tangent function, inspired by the approach of Shankar et al. [40], to transition from 2000/s up to the truncation at 8000/s. This form invokes the idea that there is gradual saturation of vWF-mediated binding at high shear. For $k_{-}(\dot{\gamma })$, we considered three, piecewise-linear forms with truncations at 2000/s, 5000/s, and 8000/s, and one using an exponential form to transition beyond 2000/s to reflect reduced bond lifetimes under elevated shear. All forms are shown in Fig 4.

The shear-dependent rate functions were constructed to capture the known nonlinear response of vWF-mediated platelet binding under flow. vWF undergoes conformational changes with increasing shear, transitioning from a compact to an extended state, beyond which further increases in shear do not substantially enhance binding kinetics [54,55]. In addition, prior modeling of GPIbα–vWF interactions suggests distinct shear regimes in bond formation kinetics, with approximately linear behavior at lower shear and a transition to enhanced binding at higher shear [56]. These observations motivate the use of piecewise and nonlinear functional forms for $k_{+}(\dot{\gamma })$ to capture both baseline and high-shear behavior.

The association rate is defined as


$$\begin{array}{c} k_{+}(\dot{\gamma })=\left\{ \begin{array}{cc} a+\frac{\dot{\gamma }-300}{1200}(b-a), & \dot{\gamma }<10000,\\ {}[6pt]a+\frac{9700}{1200}(b-a), & \dot{\gamma }\geq 10000. \end{array}\right. \end{array}$$ (11)

which preserves the approximately linear dependence observed at lower shear while introducing a nonlinear transition to enhanced binding at higher shear.

The dissociation rate $k_{-}(\dot{\gamma })$ is formulated to preserve the linear dependence observed in straight-channel studies up to approximately 2000 s^−1^, while allowing for a deviation from linear behavior at higher shear to reflect shear-dependent modulation of bond lifetimes. Accordingly, $k_{-}(\dot{\gamma })$ is defined piecewise as


$$\begin{array}{c} k_{-}(\dot{\gamma })=\left\{ \begin{array}{cc} a+\frac{\dot{\gamma }-300}{1200}(b-a), & \dot{\gamma }<2000,\\ {}[6pt]a+\frac{1700}{1200}(b-a), & \dot{\gamma }\geq 2000. \end{array}\right. \end{array}$$ (12)

which ensures continuity at $\dot{\gamma }=2000\;s^{-1}$ while capturing a slower-than-linear increase in the off-rate at elevated shear. The selection of these functional forms was guided by mechanistic considerations together with the requirement that the model reproduce occlusion times consistent with experimental observations in extravascular clotting simulations. Here, *a*, *b*, *c*, and *d* correspond to $k_{{\text{adh}}^{+}}^{\text{vWF}}$(300/s), $k_{{\text{adh}}^{+}}^{\text{vWF}}$(1500/s), $k_{{\text{adh}}^{-}}^{\text{vWF}}$(300/s), and $k_{{\text{adh}}^{-}}^{\text{vWF}}$(1500/s) for adhesion, and $k_{{\text{coh}}^{+}}^{\text{vWF}}$(300/s), $k_{{\text{coh}}^{+}}^{\text{vWF}}$(1500/s), $k_{{\text{coh}}^{-}}^{\text{vWF}}$(300/s), and $k_{{\text{coh}}^{-}}^{\text{vWF}}$(1500/s) for cohesion; estimated values of these constants are given in S2 Appendix.

### Experimental materials

Bovine serum albumin (BSA), 3,3’-dihexyloxacarbocyanine iodide (DiOC6), glutaraldehyde, 4-(2-hydroxyethyl)-1-piperazineethanesulfonic acid (HEPES), glutaraldehyde, and (3-aminopropyl)triethoxysilane (APTES) were from Sigma–Aldrich (St Louis, MO, USA). Glass luer lock syringes, 250 μL and 500 μL, were from Hamilton (Reno, NV, USA). Tridecafluoro-1,1,2,2-tetrahydrooctyltrichlorosilane (FOTS, SIT8174.0) was from Gelest (Morrisville, PA, USA). Polydimethylsiloxane base and crosslinker were from Krayden (Denver, CO, USA). Collagen related peptides CRP-XL [GCO(GPO)_10_GCOG-amide], GFOGER [GPC(GPP)_5_GFOGER(GPP)_5_GPC-amide], and VWF-III [GPC(GPP)_5_GPRGQOGVMGFO(GPP)_5_GPC-amide] were from Cambcol Laboratories (Cambridgeshire, UK). Plain glass slides (75 mm × 25 mm × 1 mm) were from Fisher Scientific (Lenexa, KS, USA). HEPES-buffered saline (HBS) was 140 mM NaCl, 1.5 mM Na_2_*HPO*_4_·2H_2_O, and 50 mM HEPES adjusted to pH 7.4. Silicon wafers of 100 mm diameter × 525 μm thickness (ID 452) were from University Wafers (South Boston, MA, USA). Photoresist polymer KMPR (1035) was from Kayaku Advanced Materials (Westborough, MA, USA). AZ 300 MIF Photoresist Developer was from Integrated Micro Materials (Argyle, TX, USA). Masterflex Microbore Transfer Tubing (Tygon ND-100–80, 0.010” ID × 0.030” OD) was from Masterflex SE (Gelsenkirchen, Germany).

### Microfluidic device fabrication

Devices were fabricated using standard soft lithography methods. In brief, room temperature KMPR 1035 was spun at 500 rpm accelerating 100 rpm/s for 10 s, then at 2300 rpm accelerating at 230 rpm/s for 30 s on a 4 inch wafer, soft baked on a hot plate at 100°C for 15 min, exposed through a transparency mask with 365 nm light at a dose of 960 mJ/cm^2^, and developed for 2–3 min. Feature dimensions were measured with an optical profilometer (VK-X3000, Keyence) to confirm feature height of 50±2 μm. Wafers with photoresist features were treated via gas deposition of FOTS under vacuum for 4 hours. PDMS was mixed at a catalyst:base ratio of 1:10, degassed, and poured over FOTS-treated wafers, and allowed to cure at 80°C. Devices were cut out of the PDMS mold, and inlet and outlet ports were punched with a 6 mm biopsy punch (504533, World Precision Instruments) and a 0.75 mm biopsy punch (504529, World Precision Instruments), respectively.

### Collagen peptide patterning

Plain glass slides were immersed in a 1:1 12N hydrochloric acid:methanol solution for 30 min, rinsed 3 times with deionized water, and dried with a nitrogen air brush. The cleaned slides were placed in an oxygen plasma cleaner (PDC-001, Harrick Plasma, Ithaca, NY, USA) at 0.3 bars of oxygen for 2 min. The slides were then immediately submerged in 1% solution of APTES for 2 min. After APTES treatment, glass slides were rinsed 3 times in deionized water and dried with compressed nitrogen, then placed on a hot plate set to 110°C for 1 min. Once cooled to room temperature, the slides were submerged in 8% glutaraldehyde for 30 min, then rinsed 3 times in deionized water and dried with a nitrogen air brush. Functionalized slides were stored in a desiccation chamber for no more than 1 week before use. A PDMS channel (*ℓ* = 50 mm, *w* = 150 μm, *h* = 50 μm) treated with FOTS via gas deposition was laid horizontally across a functionalized glass slide. CRP-XL, GFOGER, and VWF-III peptides were mixed together in 10 mM acetic acid to a final concentration of 250 μg/mL each. The microfluidic channel was filled with the peptide solution and incubated overnight at 4°C in a Parafilm-sealed petri dish with Kim-Wipes soaked in deionized water to prevent evaporation.

### Whole blood collection and preparation

Human whole blood was collected via venipuncture via 19G needle into a 5 mL vacutainer with a final concentration of 75 μM Phe-Pro-Arg-chloromethylketone PPACK (SCAT-875B-5/5, Prolytix, Essex Junction, VT, USA) and a 4 mL K_2_ EDTA BD Vacutainer (367863, Becton, Dickinson and Company, Franklin Lakes, NJ, USA). Blood cell counts were measured with a hematology analyzer (ABX Micros 60, Horiba Medical, Kyoto, Japan) in EDTA anticoagulated blood. Subjects were recruited at the University of Colorado Anschutz. PPACK anti-coagulated whole blood was incubated with 1 μM DIOC6 and anti-CD62P fluorescently labeled monoclonal antibody (1:20 v:v, 550561, Becton Dickinson, Franklin Lakes, NJ, USA). The following platelet inhibitors were added to the whole blood separately or in combination as indicated at the following concentrations: 10 mM indomethacin, 100 μM 2Me-SAMP, and 100 μM MRS2719. Platelet inhibitors were dissolved in 0.1% ethanol-HBS, and the same volume of this vehicle was added to all conditions. The whole blood with labels and inhibitors was incubated at 37°C for 10 min prior to the flow assay.

### Microfluidic blood flow assays

Microfluidic devices (*ℓ* = 50 mm, *w* = 500 μm, *h* = 50 μm) were aligned perpendicular to the collagen peptide-patterned strip, held together in a custom press, and blocked with 2% (w/v) BSA in HBS at 4°C overnight. A 50 cm length of tubing was connected to a 250 μL or 500 μL glass syringe via a 30 G × 1/2 in. Luer-lock industrial dispensing tip (CML Supply, Lexington, KY, USA). Tubing and syringe were then primed with HBS (filling approximately 10% of the syringe without air bubbles) before being connected to the outlet of the microfluidic device. The syringe was placed in a syringe pump (PHD | ULTRA, Harvard Apparatus, Holliston, MA, USA) and set to withdraw at flow rate to achieve wall shear rates 300/s or 1500/s on the bottom wall of the channel. The blood (150 μL) was placed in the device well before initiating flow. DIOC6 and PE anti-CD62P labeled platelets were detected via 494/518 nm and 555/580 nm ex/em filter sets on a spinning disc confocal microscope (Olympus IX-83 equipped with a CSU-W1 spinning disc unit, 40X, Olympus Life Science, Waltham, MA, USA) with a 15 s interval between z-stacks and a 1.13 μm interval between z-slices. Both fluorescent labels were recorded simultaneously via dual camera (ORCA-Flash4.0, Hamamatsu Photonics, Hamamatsu City, Shizuoka, Japan). Images were captured with CellSens software (Olympus Life Science, Waltham, MA, USA) as 1024 pixel × 1024 pixel with 16-bit depth.

### Image processing and data analysis

To reconstruct the platelet aggregated volumes, the VSI formatted image stacks were first imported to FIJI/ImageJ image processing software as separate TIFF files for each 16-bit channel via the Bioformats plugin. Each image stack series was then cropped to 100 μm × 100 μm or 308 pixel × 308 pixel region of interest in the center portion of the channel. We used ImageJ [57] for image processing and followed the following steps:

1. A rolling ball algorithm with a 5 pixel radius was applied to each stack to subtract background signal.
2. An automatic threshold using Otsu’s method was applied to each stack slice using the stack histogram to create a binary mask of each slice.
3. An “opening” algorithm with count = 5 was then applied to each binary mask for 5 iterations.
4. The Analyze Particles function was applied to each stack to detect distinct particles in each mask slice.

A summary table with the total area and number of particles in each slice was recorded as a CSV file. Volumes ($\mu {\text{m}}^{3}$) for each time point were then calculated by multiplying the sum of areas ($\mu {\text{m}}^{2}$) in each single-timepoint stack by the sum of height increments (1.13 μm).

## Results

### Microfluidic assays provide data to calibrate shear-dependent platelet model

**Fig 5:**
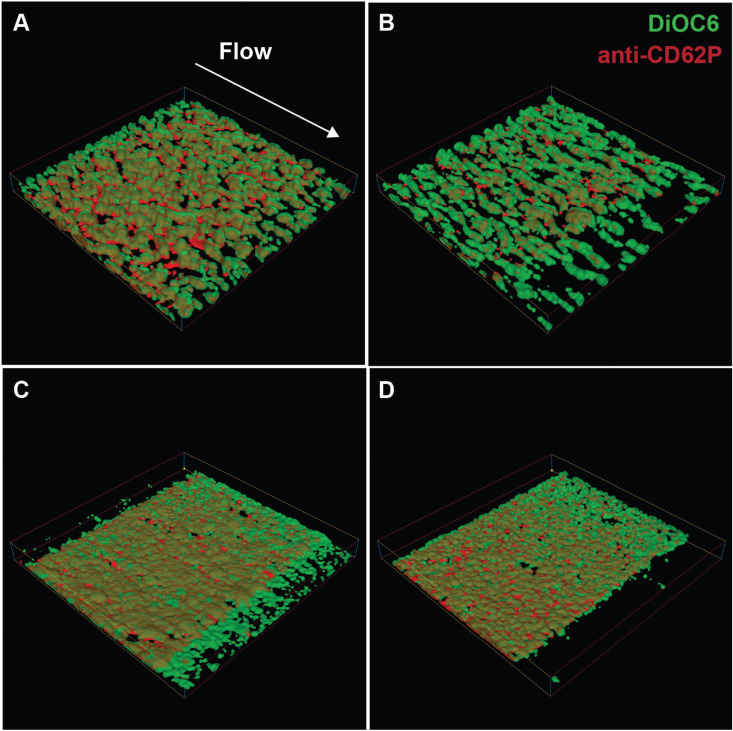
3D reconstruction of platelet aggregates from confocal microscopy. Results at 5 minutes of perfusion over collagen related peptides at 300/s (A,B) and 1500/s (C,D) with a vehicle control (A,C) or inhibition (B,D) of amplification loops (indomethacin, 2Me-SAMP, MRS2719). DiOC6 labels all platelets and anti-CD62P labels activated platelets that have secreted α-granules. Flow is from upper-left to lower-right. Dimensions of box is length = 333 μm, width = 330 μm, and height = 30 μm.

Using the microfluidic assay, we perfused PPACK anticoagulated human whole blood over collagen peptides in the presence and absence of inhibitors that block amplification by TXA_2_ and ADP. We imaged platelet aggregation under shear rates of 300/s and 1500/s shear across both conditions (see Fig 5) and tracked the kinetics of platelet accumulation with a mitochondrial membrane dye (DiOC6) and their activation state with an antibody against P-selectin, a marker of granule secretion. Platelet activation was predominantly localized to the collagen peptide surface. Initially platelet aggregates formed at 300/s are roughly symmetrical islands that grow radially, whereas at 1500/s aggregates are elongated in the direction of flow. As the surface becomes more saturated with platelets, multilayer aggregates that grow up to 12 μm away from the surface for both shear rates, while amplification loop inhibitor treated blood is confined to a few layers that are 2–3 μm in height. After 8 minutes, there are still small patches of surface not covered with aggregates at 300/s, while almost the entire surface is covered at 1500/s. These observations were obtained from n = 14 experiments at 300/s and n = 8 at 1500/s.

**Fig 6:**
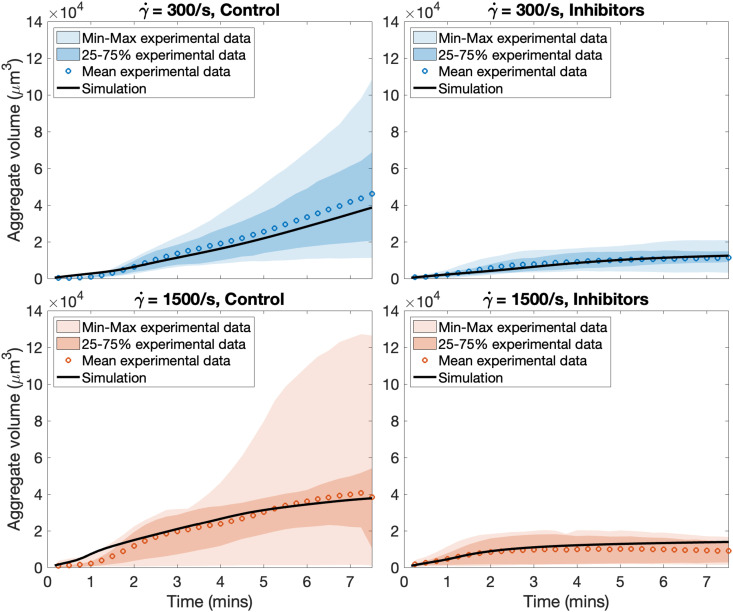
Platelet aggregate volumes in experiments and simulations. Model predictions of aggregate volume in time compared with the mean experimental data with shear rate 300/s (top row) and 1500/s (bottom row), respectively and in the absence (left column) and presence (right column) of platelet inhibitors. Darkly shaded regions consist of 50% of the data and light shaded regions plus dark regions contain 100% of the data. Open circles are the experimental data (n = 14 for shear 300/s and n = 8 for shear 1500/s) and solid lines are simulations.

To quantify these observations, we measured aggregate volume over time, shown as open circles in Fig 6, and used these data to calibrate our shear-dependent platelet aggregation model. These specific experiments allowed us to isolate and calibrate adhesion and cohesion rates in the mathematical model, independently of activation by secondary mediators such as thrombin and ADP. Simulations were performed under matched physical conditions (see S5 Appendix for details of the computational domain representing the microfluidic channel), incorporating shear-dependent functional forms for adhesion, cohesion, and activation. Donor-specific platelet counts and hematocrit and individual experimental curves are shown in S8 Appendix.

Using the calibration framework described in Methods, we identified parameter sets governing vWF-mediated adhesion, cohesion, and activation that reproduce experimental clot growth. Based on microfluidic data from the rectangular channel at a shear rate of 300/s, we calibrated adhesion, cohesion, and activation parameters, while retaining collagen adhesion and ADP-related parameters from prior work [43,45,53]. To account for shear dependence, these parameters were further refined using data at 1500/s, while all other parameters were held fixed. With these assumptions, simulated aggregate volume over time closely matched experimental mean values at both shear rates (Fig 6), demonstrating that the calibrated parameter set captures aggregate growth across shear conditions.

**Fig 7:**
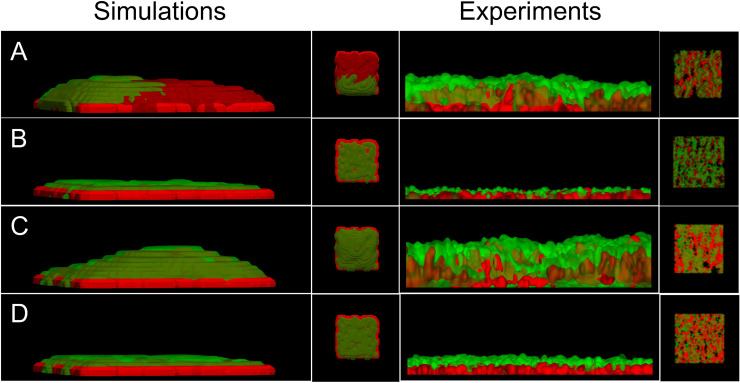
3D rendering of numerically simulated clots and experiment comparisons. Each row shows a side view (left) and a top-down view (right) of simulated (left two columns) and experimental (right two columns) vWF-mediated platelet aggregation after 450 seconds. Rows A and B correspond to simulations with an initial wall shear rate of 300 s^-1^, with and without activation by ADP, respectively. Rows C and D correspond to simulations with an initial wall shear rate of 1500 s^-1^, with and without activation by ADP, respectively. Red in the simulations represents the sum of all activated platelets and the green are bound unactivated platelets. Red and green in the experiments is as before, DiOC6 labeled and anti-CD62P (marked for granule secretion), respectively.

Adhesion and cohesion via vWF were treated as shear-dependent, and parameters were refined at shear rate 1500/s using the microfluidic data. We adapted the functional form for vWF-mediated activation from previous work [3]. Next, we assumed a linear relationship between the two shear rates, since shear varied spatially during aggregate formation, and performed simulations of platelet aggregation under flow to generate 3D renderings of the aggregates (Fig 7). We found that the simulated clots qualitatively reproduced the multilayered aggregates observed experimentally: in the absence of inhibitors, clots reached approximately 12 μm in height, whereas in their presence, they rarely extended beyond a single wall-adherent layer. Detailed parameter values and references corresponding to these simulations are provided in S2 Appendix. We performed qualitative comparisons of the spatial distribution of activated and unactivated platelets between simulations and microfluidic experiments. Isovolumes of bound unactivated platelets were plotted in green, mimicking DiOC6-labeled platelets, while activated platelets were plotted in red to reflect granule secretion detected via anti-CD62P in microfluidic experiments. In Fig 7, the sum of *P*^*bv*,*a*^, *P*^*bf*,*a*^, and *P*^*se*,*a*^ is shown in red, and *P*^*bv*,*u*^ (bound to vWF but unactivated) is shown in green. The simulations reproduce a core–shell–like organization [36,44], with the most activated platelets located near the collagen surface and a surrounding shell of unactivated platelets.

**Fig 8:**
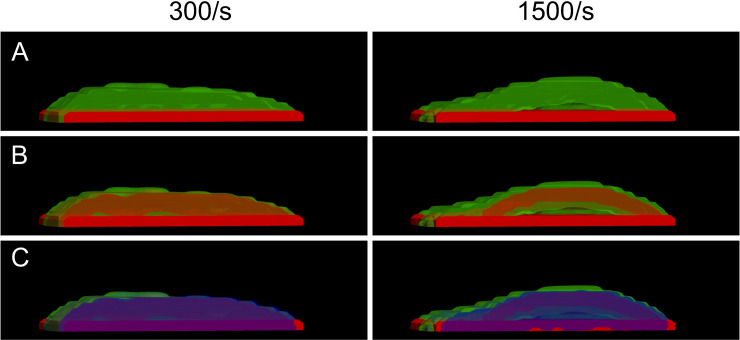
3D rendering of numerically simulated clots and intrathrombus platelet species distributions. Left column: shear rate of 300/s; right column: 1500/s. (A) Isovolume of subendothelial platelets (red) and bound unactivated platelets (green). (B) Same as in (A), with the addition of activated platelets bound via vWF (red). (C) Same as in (B), with an additional isovolume (blue) representing activated platelets bound via fibrinogen. Isovolumes are sliced along the channel centerline to enhance visualization. Together, these renderings illustrate how shear rate influences the spatial organization and composition of platelet populations within simulated thrombi.

A further strength of the mathematical model is its ability to track every platelet species and their spatial locations within the clot. To illustrate this, we separated the activated platelets into distinct isovolumes and visualized these layers in Fig 8 for the two control cases at 300/s and 1500/s. The first row shows only *P*^*se*,*a*^ and *P*^*bv*,*u*^, revealing that unactivated platelets are distributed throughout the clot. The second row adds *P*^*bv*,*a*^ (vWF-bound, activated platelets in red), showing spatial overlap with unactivated platelets. In the final row, fibrinogen-bound platelets (*P*^*bf*,*a*^) are displayed in blue, indicating that strongly cohered and activated platelets are also distributed throughout the clot. Unactivated platelets can become trapped within the clot; in the absence of thrombin and after ADP washout, these platelets remain unactivated but immobilized. Overall, the clot core contains a mixture of activated and unactivated platelets, while the outer shell is composed primarily of unactivated platelets.

### Extravascular bleeding and hemostasis in a microfluidic injury model: experiments and simulations

**Fig 9:**
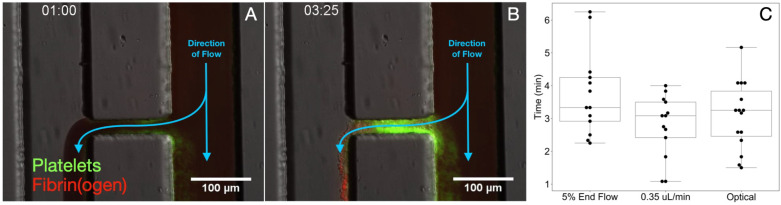
Extravascular injury geometry. Snapshots of clot formation in the bleeding chip within the injury channel: (A) after 1 minute and (B) after 3 minutes and 25 seconds. Panel (C) shows occlusion time measurements (*n* = 13) in the bleeding chip, using three different metrics [46].

To assess hemostasis in a more physiologically relevant setting with evolving shear, we transitioned from the straight-channel assays to an H-shaped “bleeding chip” that mimics blood escaping into an extravascular space (see S6 Appendix for domain specifications) [46,52,58]. Whole blood was perfused in the right “blood” channel while buffer entered the left “wash” channel at a higher flow rate, creating a pressure drop that drove blood through the central injury channel from right to left (see Fig 9). In the presence of thrombin inhibition (PPACK), platelet adhesion and aggregation were evident within one minute (Fig 9A), and full occlusion occurred at approximately 3.5 minutes (Fig 9B). Occlusion time in the bleeding-chip experiments was quantified using both optical and flow-based criteria (Fig 9C, including cessation of red blood cell passage through the injury channel and thresholds based on flow reduction. Among these, the time to reach a flow rate of 0.35 μL/min, corresponding to the median across the metrics, is used as the primary benchmark for model comparison, consistent with prior work [46].

Because the highest local shear in the extravascular injury channel can surpass 8000/s as the orifice constricts, we extended the functional forms of the on- and off-rates for vWF-mediated adhesion and cohesion beyond the calibration window in the straight channel. For simplicity, we refer to these generically as $k_{+}(\dot{\gamma })$ and $k_{-}(\dot{\gamma })$, respectively, throughout this section. We assumed that on- and off-rates for adhesion (collagen–vWF–GPIb) and cohesion (GPIb–vWF–GPIb) had the same functional forms but differed in their parameter values. We explored several different extensions of functional forms: linear, piecewise-linear, and nonlinear, each designed to capture different hypothesized behaviors under high shear. The forms we adopted are described and plotted in the Methods section. Fig 4.

**Fig 10:**
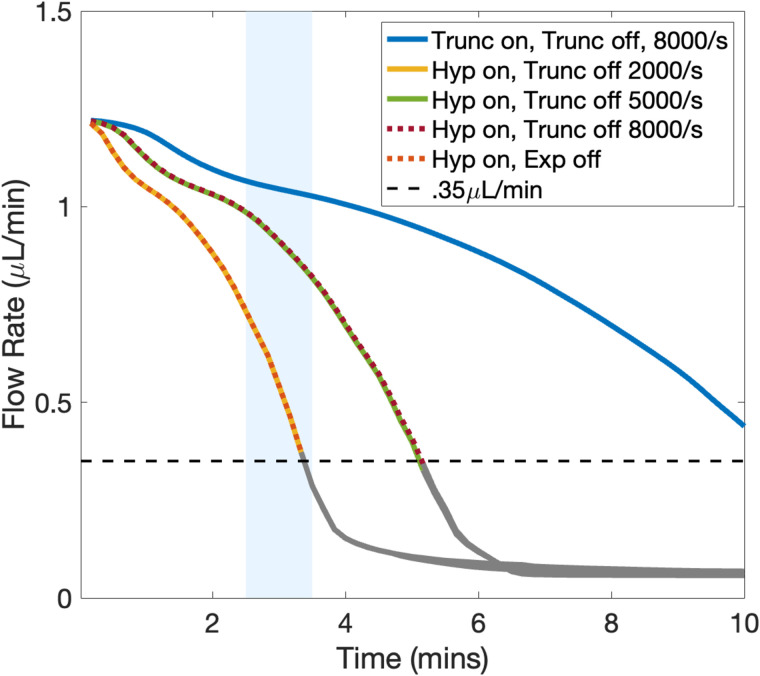
Shear-dependent adhesion and cohesion rates. Functional forms of the shear dependence for adhesion and cohesion rates involving vWF, along with the corresponding flow rates over time, measured at the end of the injury channel. The blue shaded bar indicates the interquartile range of experimental values shown in Fig 9. Flow rates below the experimentally defined occlusion threshold are shown in gray. In the legend, *Trunc* and *Hyp* denote truncated and hyperbolic functional forms, respectively, and on/off indicate whether the form was applied to the associated on- or off-rate. *Exp* denotes exponential.

We evaluated the performance of these functional by simulating clot formation in the H-shaped microfluidic geometry used in the experiments described in the previous section. Our goal was to determine whether the proposed shear-dependent rate functions could reproduce the experimentally observed injury channel flow rates used to determine occlusion times. Fig 10 shows the resulting flow rate profiles and corresponding occlusion times for several candidate forms of $k_{-}(\dot{\gamma })$.

Among the tested forms, those with off-rates truncated at $\dot{\gamma }=5000$/s and $\dot{\gamma }=8000$/s produced nearly identical results, with occlusion times lagging behind experimental observations by approximately 2–3 minutes. In contrast, the form with a plateau beginning at $\dot{\gamma }=2000$/s yielded substantially shorter occlusion times, bringing the simulations into closer agreement with the experimental range. These results indicate that maintaining relatively low off-rates (i.e., longer bond lifetimes) over the intermediate shear range of 2000/s to 8000/s is important for capturing the observed dynamics of clot formation, consistent with the need for sustained platelet cohesion under elevated shear conditions. For the on-rates, truncated forms with plateaus beginning at $\dot{\gamma }=8000$/s and $\dot{\gamma }=10000$/s resulted in occlusion times exceeding 10 minutes, substantially longer than the experimentally observed 3.5 minutes. In contrast, the hyperbolic tangent form for $k_{+}(\dot{\gamma })$ produced significantly shorter occlusion times, in agreement with experiments. For the on-rates, truncated forms with plateaus beginning at $\dot{\gamma }=8000$/s and $\dot{\gamma }=10000$/s resulted in occlusion times exceeding 10 minutes, substantially longer than the experimentally observed occlusion time of approximately 3.5 minutes. In contrast, the hyperbolic tangent form for $k_{+}(\dot{\gamma })$ produced significantly shorter occlusion times, in closer agreement with experiment. These results suggest that, over the intermediate shear range (2000/s to 8000/s), sufficiently elevated on-rates together with relatively low off-rates (i.e., longer bond lifetimes) are required to sustain platelet recruitment and cohesion. Taken together, these trends are qualitatively consistent with catch–slip bond behavior reported for vWF–GPIb interactions, in which bond lifetimes increase over an intermediate shear range before decreasing at higher shear.

### 3D visualization of clot structure across models

To investigate how shear-dependent kinetics influence clot morphology and internal structure, we first visualized the clot interiors with isovolumes of bound platelet fraction at two time points for three simulations: (i) the original *clotFoam* model without shear dependence [45], (ii) the model with hyperbolic on-rate with truncated off rate at 8000/s, and (iii) the model with hyperbolic on-rate with the exponential off rate. These simulations correspond to those shown in the previous section, where the exponential off rate produced occlusion times closest to experimental observations.

**Fig 11:**
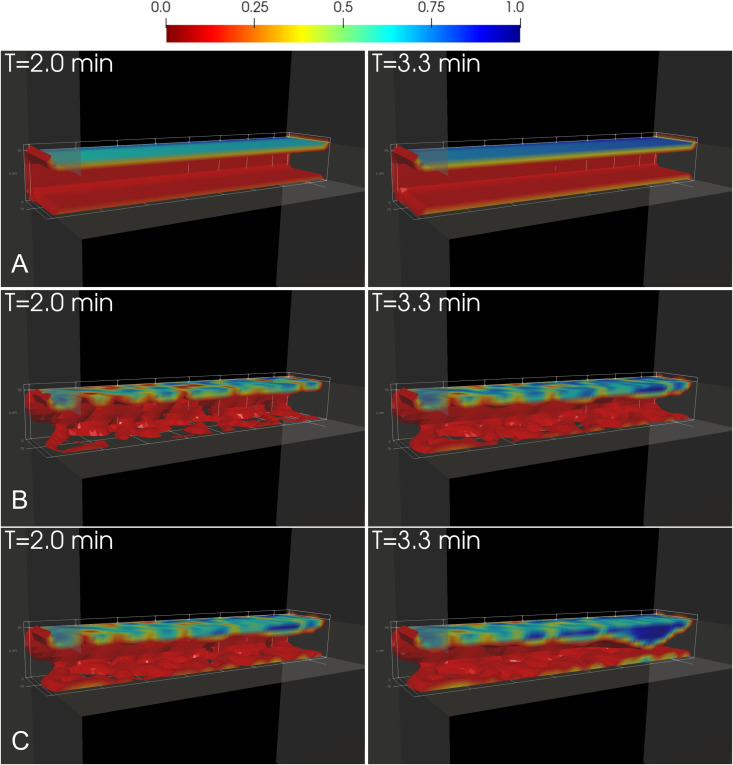
3D visualization of shear-dependent clot morphology and bound platelet distribution. Clot formation at early (left) and late (right) times without shear dependence (A) and with shear-dependent kinetics (B–C). Each clot is shown as an isovolume of bound platelet fraction, sliced along the geometry centerline to enhance visualization. Color contours on the clot surface indicate bound platelet fraction. (B) Off-rate truncated at 8000/s. (C) Exponential off-rate beginning at 2000/s. Time points are labeled.

Fig 11 shows differences in clot morphology and spatial distribution of bound platelet fraction across models. Fig 11A shows bound platelet density for shear-independent kinetics, resulting in a homogeneous clot due to constant on/off rates that produce a uniform bond lifetime. This clot did not occlude within 10 minutes. With shear-dependent kinetics (Fig 11B–C), the off rate decreases with decreasing local shear at the clot shell, allowing bonds to persist and platelet aggregation to occur. This leads to heterogeneous clot morphology. The exponential off rate (Fig 11C) produces bond lifetimes that result in occlusion times closely aligned with experimental observations.

**Fig 12:**
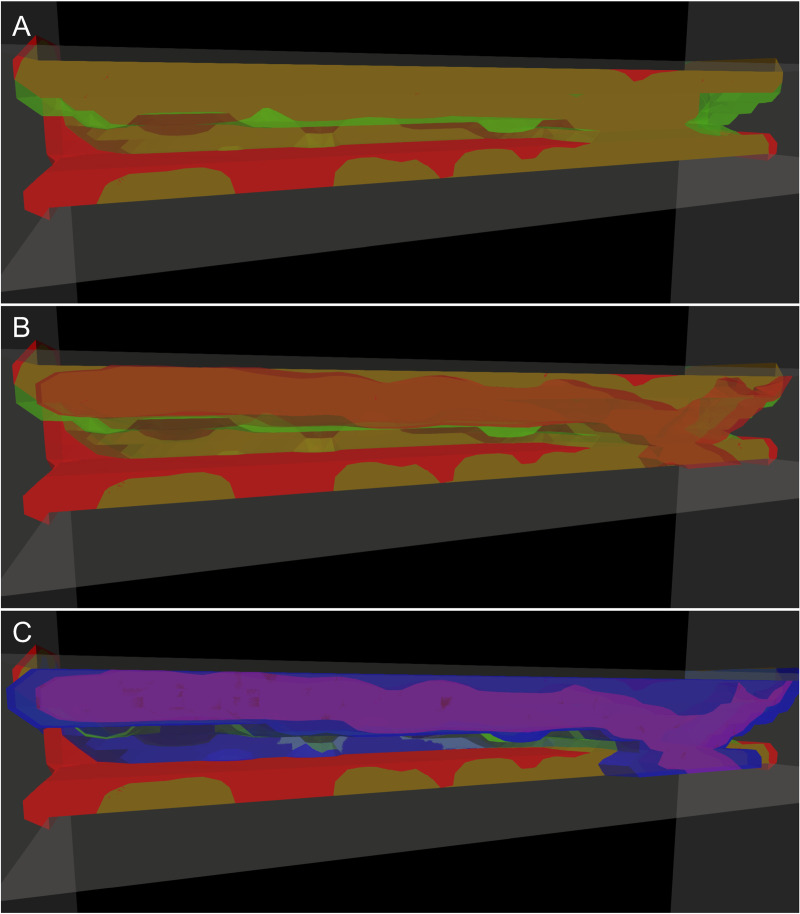
3D visualization of clot internal structure at occlusion. Clot structure at occlusion is visualized using species-resolved isovolumes, as in Fig 8. (A) Isovolumes of subendothelial platelets (red) and bound unactivated platelets (green). (B) Same as in (A), with the addition of activated platelets bound via vWF (red). (C) Same as in (B), with an additional isovolume (blue) representing activated platelets bound via fibrinogen. All isovolumes are sliced along the geometry centerline to enhance visualization. Together, these renderings illustrate the spatial organization and composition of platelet populations within the occlusion.

We next examined the internal structure of the clot by separating platelet species into distinct isovolumes, as shown in Fig 12 for the exponential off rate model. Fig 12A shows only *P*^*se*,*a*^ and *P*^*bv*,*u*^, revealing in brown the distribution of unactivated platelets throughout the clot. Fig 12B adds *P*^*bv*,*a*^ (vWF-bound, activated platelets in red). Comparing Fig 12A and B, regions where red appears within the brown indicate spatial overlap between activated and unactivated platelets, revealing their spatial organization within the clot. In Fig 12C, fibrinogen-bound platelets (*P*^*bf*,*a*^) are displayed in blue, showing that strongly cohered and activated platelets are distributed throughout the clot and contribute to occlusion. This structural organization reinforces the observations in Fig 8, where the clot core contains a mixture of activated and unactivated platelets, while the shell is composed primarily of unactivated platelets.

In S7 Appendix, we include the same structural visualizations for the shear-independent model and the shear-dependent model with the off rate truncated at 8000/s, as well as isovolumes colored by local shear rate. As the clot grows on the top wall, the local shear rate on the bottom wall remains high, and bond lifetimes in both the shear-independent model and the truncated model are insufficient to support clot development on the bottom wall, preventing occlusion.

## Discussion

We developed a platelet aggregation model that incorporates shear-dependent adhesion, activation, and cohesion dynamics within a continuum framework. This work represents a direct extension of our previously developed continuum framework [43–45,52], which models platelet transport, adhesion, and surface-mediated biochemical interactions under flow. The model was parameterized using in vitro microfluidic data designed to isolate specific platelet mechanisms under controlled shear conditions (300/s and 1500/s), and extended to higher shear regimes through biologically motivated functional forms. The extended model was subsequently evaluated in a distinct experimental geometry that mimics extravascular clot formation.

A key finding of this work is that successful reproduction of experimentally observed occlusion behavior required a balance between elevated on-rates and sufficiently low off-rates over an intermediate shear range (approximately 2000/s to 8000/s). These conditions support sustained platelet recruitment and cohesion during clot growth and are qualitatively consistent with known shear-dependent behavior of vWF-mediated interactions. Successful reproduction of occlusion times required that the on-rates increase more rapidly than a linear extrapolation of the calibrated behavior, while the off-rates increase more slowly than a linear extrapolation, over this intermediate shear range. Linear extensions of the straight-channel relationships led to insufficient platelet accumulation, whereas the adopted nonlinear forms enabled sustained aggregation and occlusion within the experimentally observed time range.

The model reproduces key structural features observed in experiments, including the core–shell architecture of thrombi and multilayer platelet accumulation. These features emerge from the coupling between shear-dependent platelet interactions and evolving local flow conditions within the growing clot, suggesting that intrathrombus heterogeneity can arise from feedback between hemodynamics and platelet adhesion dynamics.

Several limitations of this study should be noted. First, a number of kinetic parameters governing platelet-surface adhesion, cohesion, and activation were estimated. These parameters represent *effective* model quantities that capture aggregated biological processes at the platelet surface, rather than fundamental biochemical constants associated with individual molecular interactions. To constrain these parameters, we used time-resolved aggregate volume data from controlled microfluidic experiments, which provide stronger constraints than fitting to a single summary metric. Second, variability in the experimental data introduces additional uncertainty in model calibration and validation. While platelet counts and hematocrit were within the normal physiological range for all donors, prior work has shown that plasma vWF levels can vary substantially and are a major contributor to variability in platelet accumulation under flow [59]. This variability is not explicitly captured in the present model and represents an additional limitation when interpreting model predictions. Third, thrombin generation and fibrin-mediated cohesion were not included in the present simulations, as the focus of this study was to isolate shear-dependent platelet aggregation mechanisms. However, these processes are incorporated within the broader modeling framework and can be activated within the code, providing a direct path toward integrating full coagulation dynamics in future work. Finally, the model does not explicitly represent viscoelastic deformation of the platelet aggregate or the feedback of clot stresses on blood flow. The density-dependent resistance term in the momentum equations captures the impact of clot growth on local hemodynamics, but does not account for elastic stresses or clot deformation.Recent computational frameworks have incorporated viscoelastic constitutive models for blood clots to study deformation and embolization under flow [48], highlighting the importance of clot mechanics in determining stability and failure. Incorporating mechanical coupling between the clot and the fluid would require a fundamentally different modeling framework and remains an important direction for future work.

Despite these limitations, the present framework provides a computationally efficient approach for simulating thrombus growth over physiologically relevant timescales while capturing the coupled biochemical and transport processes that drive platelet aggregation. By calibrating the model in straight-channel experiments and evaluating candidate high-shear functional forms in a distinct bleeding-chip geometry, we identified a formulation that both reflects biologically plausible behavior and reproduces observed occlusion times. Importantly, the underlying continuum framework already incorporates platelet-surface coagulation reactions, providing a direct path toward integrating shear-dependent platelet aggregation with coagulation within a unified modeling framework. This work therefore represents a step toward a more comprehensive continuum model that integrates platelet aggregation and coagulation under physiologically relevant flow conditions.

## Supporting information

S1 Appendix Model Reactions and Equations. (PDF)

S2 Appendix Model Parameters. (PDF)

S3 Appendix Generation of Quasi-Random Adhesion Region. (PDF)

S4 Appendix Platelet Margination Inlet Condition. (PDF)

S5 Appendix Specifications for Simulating Microfluidic Channel. (PDF)

S6 Appendix Specifications for Simulating Extravascular Injuries. (PDF)

S7 Appendix Extravascular Clot Morphology. (PDF)

S8 Appendix Individual Donor Information. (PDF)
