# Supplementary material for: A three-dimensional shear dependent continuum model of platelet aggregation under flow
Source: PLoS Comput Biol. 2026 May 18;22(5):e1014241. doi: 10.1371/journal.pcbi.1014241 (PMC13218622; doi:10.1371/journal.pcbi.1014241)
Supplement: S1 Appendix — (PDF) [file pcbi.1014241.s001.pdf]

S1 Appendix

Platelet model reactions The following reactions describe the transitions between the various states for platelets. All parameters associated with vWF mediated binding and unbinding are dependent on the local shear rate, $\dot{\gamma}$.

Adhesion to subendothelium via vWF:

$$P^{m,u} \underset{k_{\text{adh}-}^{\text{vWF}}}{\overset{k_{\text{adh}+}^{\text{vWF}}}{\rightleftharpoons}} P^{se,u}, \tag{1}$$

$$P^{bv,u} \underset{k_{\text{adh}-}^{\text{vWF}}}{\overset{k_{\text{adh}+}^{\text{vWF}}}{\rightleftharpoons}} P^{se,u}, \tag{2}$$

$$P^{m,a} \xrightarrow{k_{\text{adh}+}^{\text{vWF}}} P^{se,a}, \tag{3}$$

Cohesion for $P^{m,u}$ via vWF:

$$P^{m,u} + P^{se,u} \underset{k_{\text{coh}-}^{\text{vWF}}}{\overset{k_{\text{coh}+}^{\text{vWF}}}{\rightleftharpoons}} P^{bv,u} + P^{se,u}, \tag{4}$$

$$P^{m,u} + P^{se,a} \underset{k_{\text{coh}-}^{\text{vWF}}}{\overset{k_{\text{coh}+}^{\text{vWF}}}{\rightleftharpoons}} P^{bv,u} + P^{se,a}, \tag{5}$$

$$P^{m,u} + P^{bv,u} \underset{k_{\text{coh}-}^{\text{vWF}}}{\overset{k_{\text{coh}+}^{\text{vWF}}}{\rightleftharpoons}} 2P^{bv,u}, \tag{6}$$

$$P^{m,u} + P^{bv,a} \underset{k_{\text{coh}-}^{\text{vWF}}}{\overset{k_{\text{coh}+}^{\text{vWF}}}{\rightleftharpoons}} P^{bv,u} + P^{bv,a}, \tag{7}$$

$$P^{m,u} + P^{bf,a} \underset{k_{\text{coh}-}^{\text{vWF}}}{\overset{k_{\text{coh}+}^{\text{vWF}}}{\rightleftharpoons}} P^{bv,u} + P^{bf,a}, \tag{8}$$

Cohesion for $P^{m,a}$ via vWF:

$$P^{m,a} + P^{se,u} \underset{k_{\text{coh}-}^{\text{vWF}}}{\overset{k_{\text{coh}+}^{\text{vWF}}}{\rightleftharpoons}} P^{bv,a} + P^{se,u}, \tag{9}$$

$$P^{m,a} + P^{se,a} \underset{k_{\text{coh}-}^{\text{vWF}}}{\overset{k_{\text{coh}+}^{\text{vWF}}}{\rightleftharpoons}} P^{bv,a} + P^{se,a}, \tag{10}$$

$$P^{m,a} + P^{bv,u} \underset{k_{\text{coh}-}^{\text{vWF}}}{\overset{k_{\text{coh}+}^{\text{vWF}}}{\rightleftharpoons}} P^{bv,a} + P^{bv,u}, \tag{11}$$

$$P^{m,a} + P^{bv,a} \underset{k_{\text{coh}-}^{\text{vWF}}}{\overset{k_{\text{coh}+}^{\text{vWF}}}{\rightleftharpoons}} 2P^{bv,a}, \tag{12}$$

$$P^{m,a} + P^{bf,a} \underset{k_{\text{coh}-}^{\text{vWF}}}{\overset{k_{\text{coh}+}^{\text{vWF}}}{\rightleftharpoons}} P^{bv,a} + P^{bf,a}, \tag{13}$$

Fibrin(ogen) mediated binding:

$$P^{m,a} + P^{bv,a} \xrightarrow{k_{\text{coh}}^{\text{fbg}}} 2P^{bf,a}, \tag{14}$$

$$P^{m,a} + P^{bf,a} \xrightarrow{k_{\text{coh}}^{\text{fbg}}} 2P^{bf,a} \tag{15}$$

$$P^{m,a} + P^{se,a} \xrightarrow{k_{\text{coh}}^{\text{fbg}}} P^{bf,a} + P^{se,a}, \tag{16}$$

$$P^{bv,a} + P^{bv,a} \xrightarrow{k_{\text{coh}}^{\text{fbg}}} 2P^{bf,a}, \tag{17}$$

$$P^{bv,a} + P^{bf,a} \xrightarrow{k_{\text{coh}}^{\text{fbg}}} 2P^{bf,a}, \tag{18}$$

$$P^{bv,a} + P^{se,a} \xrightarrow{k_{\text{coh}}^{\text{fbg}}} P^{bf,a} + P^{se,a}, \tag{19}$$

Activation by high shear:

$$P^{bv,u} \xrightarrow{k_{\text{act}}^{\text{vWF}}} P^{bv,a}, \tag{20}$$

$$P^{se,u} \xrightarrow{k_{\text{act}}^{\text{vWF}}} P^{se,a}, \tag{21}$$

Activation by ADP:

$$P^{m,u} \xrightarrow{k_{\text{act}}^{\text{ADP}}} P^{m,a}, \tag{22}$$

$$P^{bv,u} \xrightarrow{k_{\text{act}}^{\text{ADP}}} P^{bv,a}, \tag{23}$$

$$P^{se,u} \xrightarrow{k_{\text{act}}^{\text{ADP}}} P^{se,a}, \tag{24}$$

Activation/adhesion by collagen:

$$P^{se,u} \xrightarrow{k_{\text{act}}^{\text{col}}} P^{se,a}, \tag{25}$$

$$P^{bv,a} \xrightarrow{k_{\text{adh}}^{\text{col}}} P^{se,a}, \tag{26}$$

$$P^{bf,a} \xrightarrow{k_{\text{adh}}^{\text{col}}} P^{se,a}. \tag{27}$$

Platelet model equations The seven partial differential equations that govern platelet aggregation are:

$$\begin{aligned}
\frac{\partial P^{m,u}}{\partial t} = &- \underbrace{\boldsymbol{\nabla} \cdot \{W(\theta^T)(\vec{\mathbf{u}} P^{m,u} - D_P \boldsymbol{\nabla} P^{m,u})\}}_{\text{Transport via advection and diffusion}} \\
&- \underbrace{k_{\text{adh}^+}^{\text{vWF}}(\dot{\gamma}) H_{\text{adh}}(\vec{\mathbf{x}}) P_{\max}(1-\theta^B) P^{m,u}}_{\text{Adhesion to subendothelium via vWF}} \quad + \quad \underbrace{k_{\text{adh}^-}^{\text{vWF}}(\dot{\gamma}) P^{se,u}}_{\text{Unbinding due to shear}} \\
&- \underbrace{k_{\text{coh}^+}^{\text{vWF}}(\dot{\gamma}) \Big[g(\eta^U) + g(\eta^A) \Big] P_{\max} P^{m,u}}_{\text{Cohesion with bound species via vWF}} \ + \ \underbrace{k_{\text{coh}^-}^{\text{vWF}}(\dot{\gamma}) P^{bv,u}}_{\text{Unbinding due to shear}} \\
&- \underbrace{A_{\text{ADP}}([\text{ADP}]) P^{m,u}}_{\text{Activation by ADP}},
\end{aligned} \tag{28}$$

$$\begin{aligned}
\frac{\partial P^{m,a}}{\partial t} = &- \boldsymbol{\nabla} \cdot \{W(\theta^T)(\vec{\mathbf{u}} P^{m,a} - D_P \boldsymbol{\nabla} P^{m,a})\} \\
&- k_{\text{adh}^+}^{\text{vWF}}(\dot{\gamma}) H_{\text{adh}}(\vec{\mathbf{x}}) P_{\max}(1-\theta^B) P^{m,a} \\
&- k_{\text{coh}^+}^{\text{vWF}}(\dot{\gamma}) \Big[g(\eta^U) + g(\eta^A) \Big] P_{\max} P^{m,a} \ - \ \underbrace{k_{\text{coh}}^{\text{fbg}} g(\eta^A) P_{\max} P^{m,a}}_{\text{Cohesion via fibrinogen}} \\
&+ k_{\text{coh}^-}^{\text{vWF}}(\dot{\gamma}) P^{bv,a} \ + \ A_{\text{ADP}}([\text{ADP}]) P^{m,u},
\end{aligned} \tag{29}$$

$$\frac{\partial P^{bv,u}}{\partial t} = -k_{\text{adh}^+}^{\text{vWF}}(\dot{\gamma}) H_{\text{adh}}(\vec{\mathbf{x}}) P_{\max}(1-\theta^B) P^{bv,u}$$

$$
\begin{aligned}
&+ \underbrace{k_{\text{coh}^+}^{\text{vWF}}(\dot{\gamma})\Big[g(\eta^U)+g(\eta^A)\Big]P_{\max}P^{m,u}}_{P^{m,u}\text{ cohesion via vWF}} \;-\; k_{\text{coh}^-}^{\text{vWF}}(\dot{\gamma})P^{bv,u} \\
&- A_{\text{ADP}}([\text{ADP}])P^{bv,u} \;-\; \underbrace{k_{\text{act}}^{\text{vWF}}(\dot{\gamma})P^{bv,u}}_{\text{Activation by shear}} \qquad (30)
\end{aligned}
$$

$$
\begin{aligned}
\frac{\partial P^{bv,a}}{\partial t} &= -k_{\text{adh}}^{\text{col}}H_{\text{adh}}(\vec{\mathbf{x}})P_{\max}(1-\theta^B)P^{bv,a} \\
&+ \underbrace{k_{\text{coh}^+}^{\text{vWF}}(\dot{\gamma})\Big[g(\eta^U)+g(\eta^A)\Big]P_{\max}P^{m,a}}_{P^{m,a}\text{ cohesion via vWF}} \;-\; k_{\text{coh}^-}^{\text{vWF}}(\dot{\gamma})P^{bv,a} \\
&- k_{\text{coh}}^{\text{fbg}}g(\eta^A)P_{\max}P^{bv,a} \;+\; A_{\text{ADP}}([\text{ADP}])P^{bv,u} \;+\; k_{\text{act}}^{\text{vWF}}(\dot{\gamma})P^{bv,u} \qquad (31)
\end{aligned}
$$

$$
\frac{\partial P^{bf,a}}{\partial t} = -k_{\text{adh}}^{\text{col}}H_{\text{adh}}(\vec{\mathbf{x}})P_{\max}(1-\theta^B)P^{bf,a} \;+\; k_{\text{coh}}^{\text{fbg}}g(\eta^A)P_{\max}\Big(P^{m,a}+P^{bv,a}\Big), \qquad (32)
$$

$$
\begin{aligned}
\frac{\partial P^{se,u}}{\partial t} &= \underbrace{k_{\text{adh}^+}^{\text{vWF}}(\dot{\gamma})H_{\text{adh}}(\vec{\mathbf{x}})P_{\max}(1-\theta^B)(P^{m,u}+P^{bv,u}) \;-\; k_{\text{adh}^-}^{\text{vWF}}(\dot{\gamma})P^{se,u}}_{P^{m,u}\text{ and }P^{bv,u}\text{ binding/unbinding to subendothelium via vWF}} \\
&- A_{\text{ADP}}([\text{ADP}])P^{se,u} \;-\; k_{\text{act}}^{\text{vWF}}(\dot{\gamma})P^{se,u} \;-\; \underbrace{k_{\text{act}}^{\text{col}}P^{se,u}}_{\text{Activation by collagen}}, \qquad (33)
\end{aligned}
$$

$$
\begin{aligned}
\frac{\partial P^{se,a}}{\partial t} &= k_{\text{adh}^+}^{\text{vWF}}(\dot{\gamma})H_{\text{adh}}(\vec{\mathbf{x}})P_{\max}(1-\theta^B)P^{m,a} \\
&+ k_{\text{adh}}^{\text{col}}H_{\text{adh}}(\vec{\mathbf{x}})(P_{\max}-P^{se,u}-P^{se,a})(P^{bv,a}+P^{bf,a}) \\
&+ A_{\text{ADP}}([\text{ADP}])P^{se,u} \;+\; k_{\text{act}}^{\text{vWF}}(\dot{\gamma})P^{se,u} \;+\; k_{\text{act}}^{\text{col}}P^{se,u}. \qquad (34)
\end{aligned}
$$

ADP secretion and transport

$$
\frac{\partial \text{ADP}}{\partial t} = -\boldsymbol{\nabla}\cdot\{\vec{\mathbf{u}}\text{ADP} - D_{\text{ADP}}\boldsymbol{\nabla}\text{ADP}\} + \sigma_{\text{release}}(\vec{\mathbf{x}},t), \qquad (35)
$$

$$
\sigma_{\text{release}}(\vec{\mathbf{x}},t) = \int_0^\infty \hat{A}R(\tau)\frac{\partial}{\partial t}\left(P^{se,a}+P^{bv,a}+P^{bf,a}\right)d\tau. \qquad (36)
$$
