# Supplementary material for: A three-dimensional shear dependent continuum model of platelet aggregation under flow
Source: PLoS Comput Biol. 2026 May 18;22(5):e1014241. doi: 10.1371/journal.pcbi.1014241 (PMC13218622; doi:10.1371/journal.pcbi.1014241)
Supplement: S2 Appendix — (PDF) [file pcbi.1014241.s002.pdf]

S2 Appendix

Model parameters

Table A. Fluid equation parameters.

Name	Value	Units	Notes
Fluid density (ρ)	1.0×10^{-3}	g/mm^3	Danes & Leiderman [1]
Dynamic viscosity (μ)	2.62507×10^{-3}	g/mm/s	Danes & Leiderman [1]
Kinematic viscosity (ν)	2.62507	mm^2/s	$\nu = \mu/\rho$
Carman-Kozeny constant (C_{CK})	1.0×10^6	mm^2	Danes & Leiderman [1]

Table B. Diffusion coefficients for platelets and ADP.

Name	Value	Units	Notes
Platelets (D_P)	2.5×10^{-5}	mm^2/s	Turrito & Leonard [2]
ADP (D_{ADP})	5×10^{-4}	mm^2/s	Grabowski et al. [3]

Table C. Platelet characteristic parameters.

Name	Value	Units	Notes
Platelet diameter (P_{diam})	3×10^{-3}	mm	Leiderman & Fogelson [4]
Maximum packing density (P_{max})	6.67×10^7	$plts/mm^3$	Leiderman & Fogelson [4]
Normal density of platelets (P_0)	2.5×10^5	$plts/mm^3$	Weiss [5]

Table D. Binding affinity parameters.

Name	Value	Units	Notes
Threshold for binding affinity (η_t)	1.0×10^{-1}	1	Leiderman & Fogelson [4]
Rapid change in binding affinity ($\eta^* + \eta_t$)	$0.5 - \eta_t$	1	Leiderman & Fogelson [4]
Length of diffusion for the virtual substance (L_η)	$2.0 \times P_{\text{diam}}$	1	Montgomery et al. [6]

Table E. Shear rate dependent adhesion and cohesion parameters for 3D straight channel. The functional forms interpolate the associated low and high shear kinetic rates, $k^{\text{val}}_{\dot\gamma_{\text{low}}}$ and $k^{\text{val}}_{\dot\gamma_{\text{high}}}$, respectively, where $\dot\gamma_{\text{low}} = 300$ s^{-1} and $\dot\gamma_{\text{high}} = 1500$ s^{-1}. The exact functional forms are provided at the bottom of the table and shown in Fig. A.

Rate	Value at $\dot\gamma_{\text{low}}$	Value at $\dot\gamma_{\text{high}}$	Units	Notes
$k^{\text{vWF}}_{\text{adh}^+}(\dot\gamma)$	2.9891×10^{-8}	8.7181×10^{-8}	mm^3/s	On-rate for adhesion via vWF.
$k^{\text{vWF}}_{\text{adh}^-}(\dot\gamma)$	0.47	1.6	1/s	Off-rate for adhesion via vWF.
$k^{\text{vWF}}_{\text{coh}^+}(\dot\gamma) \times P_{\max}$	9.0×10^3	1.875×10^4	1/s	On-rate for cohesion via vWF.
$k^{\text{vWF}}_{\text{coh}^-}(\dot\gamma)$	3.75×10^2	5.0×10^2	1/s	Off-rate for cohesion via vWF.
$k^{\text{vWF}}_{+}(\dot\gamma)$	$\min\left\{k^{\text{vWF}}_{-}(\dot\gamma),\ k^{\text{vWF}}_{-}(\dot\gamma_{\max})\right\}$			Functional form for shear-dependent on-rates.<br>The $\max\left\{k^{\text{vWF}}_{+}(\dot\gamma)\right\}$ occurs at $\dot\gamma_{\max} =$ 10,000 s^{-1}.
$k^{\text{vWF}}_{-}(\dot\gamma)$	$k^{\text{val}}_{\dot\gamma_{\text{low}}} + \frac{\dot\gamma - \dot\gamma_{\text{low}}}{\dot\gamma_{\text{high}} - \dot\gamma_{\text{low}}}\left(k^{\text{val}}_{\dot\gamma_{\text{high}}} - k^{\text{val}}_{\dot\gamma_{\text{low}}}\right)$			Functional form for shear-dependent off-rates.

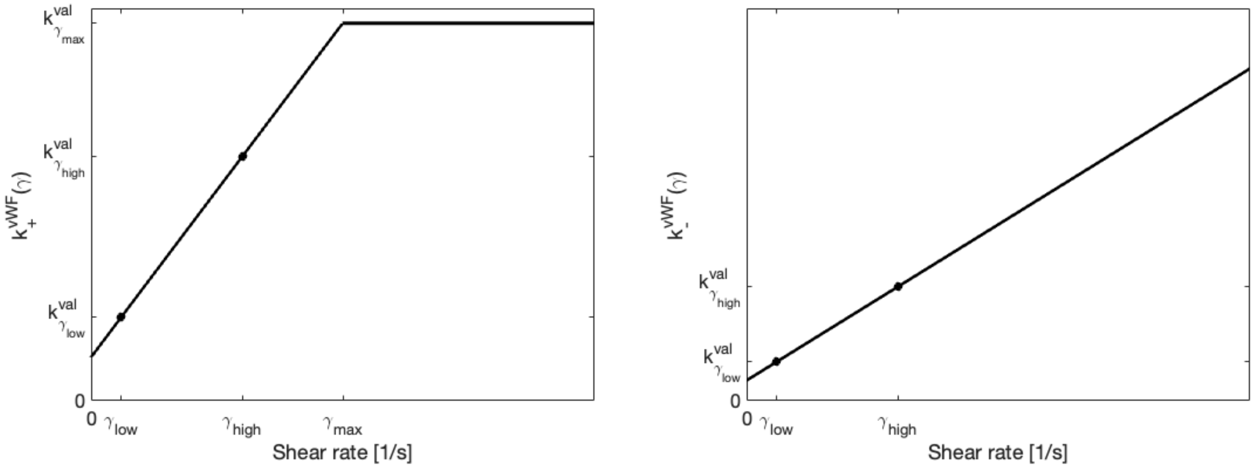

Fig A. Functional form for vWF mediated adhesion/cohesion rates used in 3D straight channel. Left: the form for the shear-dependent on-rates. Right: the form for the shear dependent off-rates.

Table F. Constant adhesion and cohesion parameters.

Rate	Value	Units	Notes
$k^{\text{col}}_{\text{adh}}$	3.3212×10^{-8}	mm^3/s	Rate of adhesion by collagen. Converted to mm^3/s from Kuharsky & Fogelson [7].
$k^{\text{fbg}}_{\text{coh}}$	5.0×10^3	1/s	Rate of cohesion via $\alpha_{IIb}\beta_3$ receptors. Numerically estimated.

Table G. Activation parameters.

Rate	Value	Units	Notes
$k_{\text{act,r}}^{\text{vWF}}$	3.4×10^{-1}	1/s	Rate of activation from shear stress between GPIb receptors and vWF. Numerically estimated.
$k_{\text{act}}^{\text{vWF}}(\dot{\gamma})$	$\frac{k_{\text{act,r}}^{\text{vWF}}}{2}\left\{1+\tanh\left(\sigma_{\text{act}}\frac{(\dot{\gamma}-\dot{\gamma}_{\text{act}}^{c})}{\dot{\gamma}_{\text{act}}^{c}}\right)\right\}$	1/s	Functional form for activation due to shear stress. The critical shear rate $\dot{\gamma}_{\text{act}}^{c} = 5000\ \text{s}^{-1}$ has been observed in the literature [8, 9, 10] and the shape of the function is consistent with the function used by Zhussupbekov et al. [11] that describes the unfolding rate of vWF. The steepness parameter is $\sigma_{\text{act}} = 2.5$.
$k_{\text{act}}^{\text{col}}$	3.3×10^{-1}	1/s	Rate of activation by collagen embeded in subendothelium. Numerically estimated.
$k_{\text{act}}^{\text{ADP}}$	3.4×10^{-1}	1/s	Rate of activation by ADP. Leiderman & Fogelson [4].

Table H. ADP related parameters and functions.

Name	Value	Units	Notes
$A_{\text{ADP}}([\text{ADP}])$	$\frac{k_{\text{act}}^{\text{ADP}}\left([\text{ADP}]-s_{\text{ADP}}\right)}{[\text{ADP}]^{*}+[\text{ADP}]-s_{\text{ADP}}}$	1/s	Modified Hill function that describes platelet activation due to ADP.
$[\text{ADP}]^{*}$	1.0×10^{-4}	nmol/mm^3	Decreased from Leiderman & Fogelson [4] to increase model sensitivity to low concentrations of ADP.
s_{ADP}	1.0×10^{-5}	nmol/mm^3	Parameter that shifts Hill function to avoid unwanted activation away from thrombus.
$\hat{A}$	2.0×10^{-3}	nmol/mm^3	Total ADP released by an activated platelet [4].
τ_F	6	s	Total time of ADP secretion [4, 6].
$\Delta\tau$	0.25	s	Step size for calculating ADP secretion σ_{release} [4, 6].

References

[1] Danes NA, Leiderman K. A density-dependent FEM-FCT algorithm with application to modeling platelet aggregation. International journal for numerical methods in biomedical engineering. 2019;35(9):e3212. doi:10.1002/cnm.3212.

[2] Turitto VT, Leonard EF. Platelet adhesion to a spinning surface. ASAIO Journal. 1972;18(1):348–354.

[3] Grabowski EF, Franta JT, Didisheim P. Platelet aggregation in flowing blood in vitro. II. Dependence of aggregate growth rate on ADP concentration and shear rate. Microvascular Research. 1978;16(2):183–195. doi:10.1016/0026-2862(78)90054-7.

[4] Leiderman K, Fogelson AL. Grow with the flow: a spatial–temporal model of platelet deposition and blood coagulation under flow. Mathematical medicine and biology: a journal of the IMA. 2011;28(1):47–84. doi:10.1093/imammb/dqq005.

[5] Weiss HJ. Platelet Physiology and Abnormalities of Platelet Function: (First of Two Parts). New England Journal of Medicine. 1975;293(11):531–541. doi:10.1056/NEJM197509112931105.

[6] Montgomery D, Municchi F, Leiderman K. clotFoam: An open-source framework to simulate blood clot formation under arterial flow. SoftwareX. 2023;23:101483. doi:10.1016/j.softx.2023.101483.

[7] Kuharsky AL, Fogelson AL. Surface-Mediated Control of Blood Coagulation: The Role of Binding Site Densities and Platelet Deposition. Biophysical journal. 2001;80(3):1050–1074.

[8] Alexander-Katz A, Schneider M, Schneider S, Wixforth A, Netz R. Shear-flow-induced unfolding of polymeric globules. Physical review letters. 2006;97(13):138101.

[9] Schneider S, Nuschele S, Wixforth A, Gorzelanny C, Alexander-Katz A, Netz R, et al. Shear-induced unfolding triggers adhesion of von Willebrand factor fibers. Proceedings of the National Academy of Sciences. 2007;104(19):7899–7903. doi:10.1073/pnas.0608422104.

[10] Lippok S, Radtke M, Obser T, Kleemeier L, Schneppenheim R, Budde U, et al. Shear-induced unfolding and enzymatic cleavage of full-length VWF multimers. Biophysical journal. 2016;110(3):545–554. doi:10.1016/j.bpj.2015.12.023.

[11] Zhussupbekov M, Rojano RM, Wu WT, Antaki JF. A Continuum Model for the Unfolding of von Willebrand Factor. Annals of Biomedical Engineering. 2021;49(9):2646–2658. doi:10.1007/s10439-021-02845-5.
