# Supplementary material for: A three-dimensional shear dependent continuum model of platelet aggregation under flow
Source: PLoS Comput Biol. 2026 May 18;22(5):e1014241. doi: 10.1371/journal.pcbi.1014241 (PMC13218622; doi:10.1371/journal.pcbi.1014241)
Supplement: S3 Appendix — (PDF) [file pcbi.1014241.s003.pdf]

S3 Appendix

Generation of quasi-random adhesion region

To achieve quasi-random clot growth, the adhesion region, $H_{\text{adh}}(\vec{\mathbf{x}})$, is patterned with Gaussian's, $g_n(\vec{\mathbf{x}})$, centered at Halton points [1], $(x_{c,n},\ z_{c,n})$, on the x-z plane. The Halton sequence generates sequences of spatial points that are maximally self-avoiding. Provided a computational domain $\Omega = [x_0,\ x_{\max}] \times [y_0,\ y_{\max}] \times [z_0,\ z_{\max}]$, the adhesion region defined as:

$$H_{\text{adh}}(x, y, z) = \min\left\{1,\ \sum_{n=1}^{N_p} g_n(x, y, z)\right\}, \tag{1}$$

where N_P is the number Gaussians. The nth Gausssian centered at $(x_{c,n}, y_0,\ z_{c,n})$ is defined by

$$g_n(x, y, z) = \exp\left\{-\frac{(x - x_{c,n})^2}{2\sigma_x^2} - \frac{(y - y_0)^2}{2\sigma_y^2} - \frac{(z - z_{c,n})^2}{2\sigma_z^2}\right\}. \tag{2}$$

with standard deviations $(\sigma_x,\ \sigma_y,\ \sigma_z)$. The Halton points in the x-direction, $x_{c,n}$, are generated with a base of 2, while the points in the z-direction, $z_{c,n}$ are generated with a base of 3.

Simulations with various adhesion regions produce slight differences in aggregate volume as shown in Fig. A. The various adhesion regions are shown in Fig. B. Each region is defined so that the average value is:

$$\bar{H}_{\text{adh}} = \text{mean}(H_{\text{adh}}(\vec{\mathbf{x}})) \approx 0.3632, \quad \text{for } \vec{\mathbf{x}} \in \Omega_{\text{inj}}.$$

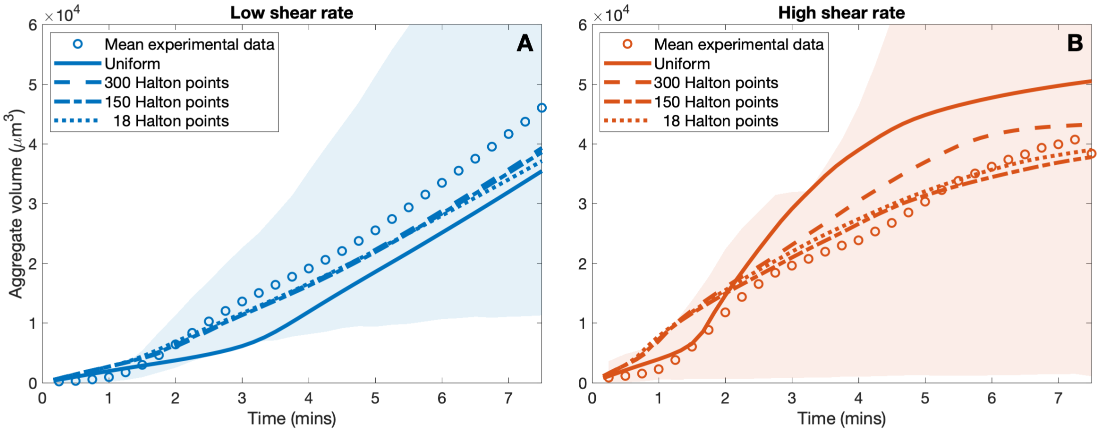

Fig A. Sensitivity to adhesion region H_{adh}.

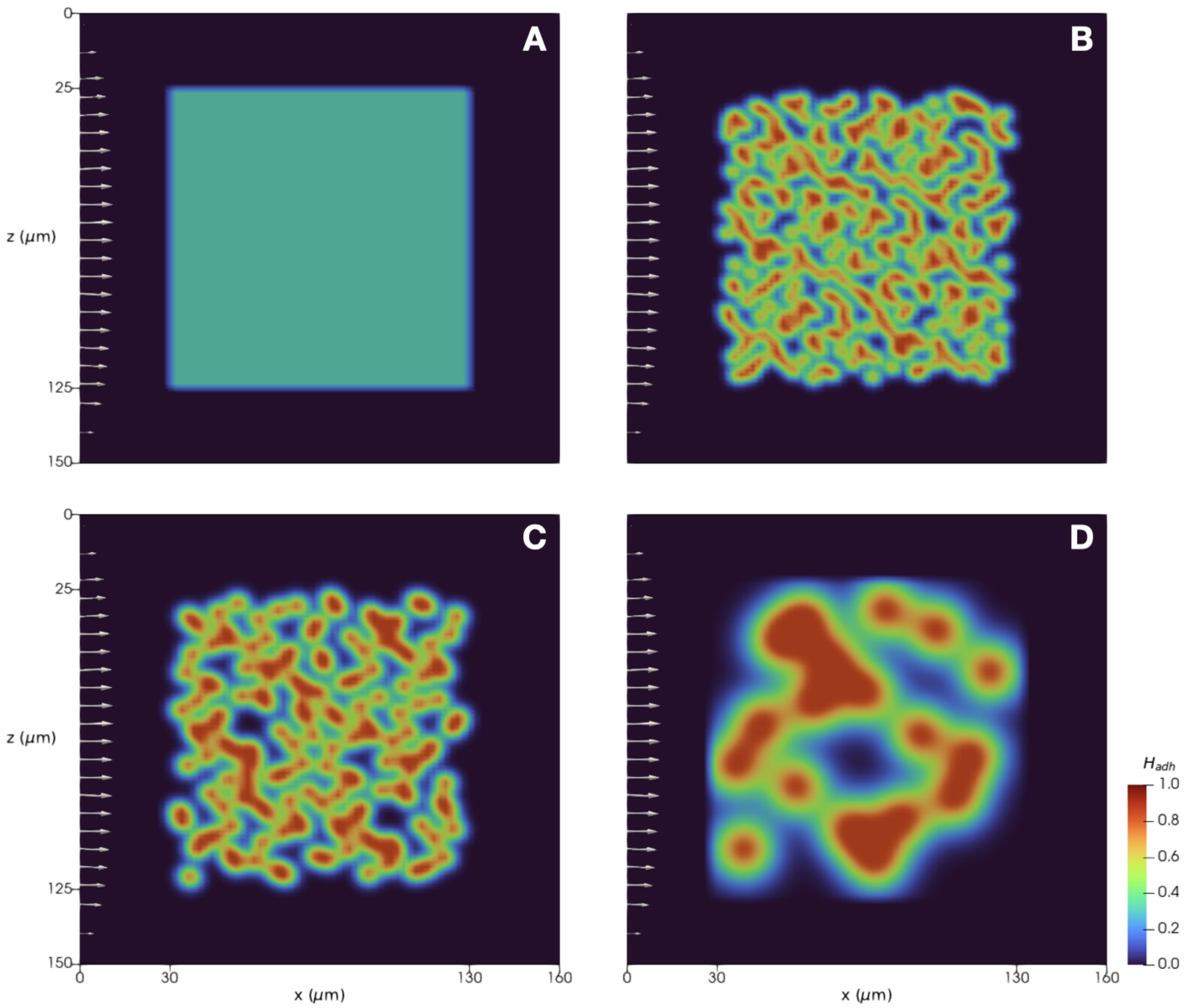

Fig B. Adhesion regions used in sensitivity analysis. Top down views of the adhesion regions, H_{adh}. The arrows indicate the direction of flow from the inlet. Each H_{adh} has an average value of approximately 0.3632. The three adhesion regions with Halton points have $\sigma_y = 3 \times P_{\text{diam}}$. A: a uniform adhesion region. B: 300 Halton points with $\sigma_x = \sigma_z = 0.56475 \times P_{\text{diam}}$. C: 150 Halton points with $\sigma_x = \sigma_z = 0.8 \times P_{\text{diam}}$. D: 18 Halton points with $\sigma_x = \sigma_z = 2.3325 \times P_{\text{diam}}$.

References

[1] Halton JH. On the efficiency of certain quasi-random sequences of points in evaluating multi-dimensional integrals. Numerische Mathematik. 1960;2:84–90. doi:10.1007/bf01386213.
