# Supplementary material for: A three-dimensional shear dependent continuum model of platelet aggregation under flow
Source: PLoS Comput Biol. 2026 May 18;22(5):e1014241. doi: 10.1371/journal.pcbi.1014241 (PMC13218622; doi:10.1371/journal.pcbi.1014241)
Supplement: S4 Appendix — (PDF) [file pcbi.1014241.s004.pdf]

# S4 Appendix

## Platelet margination inlet condition

Provided a rectangular channel domain defined by $\Omega = [x_0,\ x_{\text{max}}] \times [y_0,\ y_{\text{max}}] \times [z_0,\ z_{\text{max}}]$, the inlet profile for mobile-unactivated platelets entering the domain at $x = x_0$, is given by:

$$P^{m,u}(x_0, y, z, t) = P_0 c(y, z), \tag{1}$$

where $P_0$ is the normal density of platelets. The normalized shape function $c(y, z)$ is adapted from Eckstein and Belgacem [1] and defined by:

$$c(y, z) = C_0\, s(y, z), \tag{2}$$

where $C_0$ is a normalizing parameter. Assuming that the shear rate on the top/bottom of the channel is higher than the sides of the channel, the shape function is:

$$s(y, z) = \min\left(\tilde{s}(y, z), \tilde{s}(y, z_{\text{mid}})\right), \tag{3}$$

where $z_{\text{mid}} = (z_{\text{max}} - z_0)/2$ and

$$\tilde{s}(y, z) = 1 + K_y R(y, r_y)^{m-1}(1 - R(y, r_y))^{n-1} + K_z R(z, r_z)^{m-1}(1 - R(z, r_z))^{n-1}. \tag{4}$$

This definition of the shape function $s(y, z)$ ensures the desired peak-to-center ratio and limits unwanted excess of platelets in the corners of the channel.

The amplitude parameters $K_y$ and $K_z$ depend on the local shear rate, $\dot{\gamma}$, on the top/bottom and front/back of the channel, respectively. The values of the amplitude parameters are calculated using:

$$K(\dot{\gamma}) = c_0 + c_1\dot{\gamma} + c_2\dot{\gamma}^2, \tag{5}$$

where $c_0 = 71$, $c_1 = 0.265$ s and $c_2 = -6.0 \times 10^{-6}$ s$^2$, which come from fitting the amplitude parameters used in Leiderman and Fogelson [2] for shear rates $\gamma$ =500, 1000, 1500 s$^{-1}$, with a second degree polynomial via least squares.

The $R$ function is defined by:

$$R(\xi, r_\xi) = \frac{|\xi - r_\xi|}{r_\xi}, \tag{6}$$

with $r_\xi = (\xi_{\text{max}} - \xi_0)/2$. The exponents are set to $m = 19$ and $n = 2$, and the normalization parameter is defined by

$$\frac{1}{C_0} = \frac{1}{A}\int_{z_0}^{z_{\text{max}}}\int_{y_0}^{y_{\text{max}}} s(y, z)\, dy\, dz, \tag{7}$$

with an inlet surface area of $A = [y_{\text{max}} - y_0] \times [z_{\text{max}} - z_0]$. Once the shape function $c(y, z)$ has been calculated, it is scaled by the normal density of platelets $P_0$ as in Eq. (1), resulting in the inlet profile for the mobile-unactivated platelets. The platelet profiles used for the low and high shear experiments are shown in Fig. A, where $y_0 = z_0 =$ $\mu$m, $y_{\text{max}} = 50$ $\mu$m and $z_{\text{max}} = 150$ $\mu$m. The wall-shear rates and $K_y$, $K_z$ values are listed in Tables B and A, respectively.

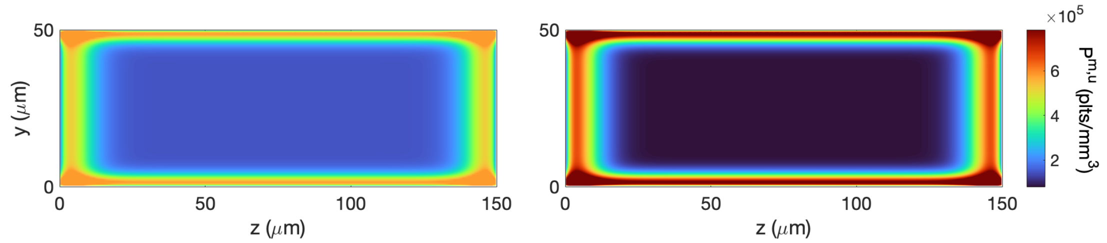


**Fig A.** The marginated platelet profiles for low shear (left) and high shear (right) experiments.

**Table A. Shear rates used to calculate amplitude parameters.**

| | Top/bottom | Sides |
|---|---|---|
| Low shear experiments | 300 $s^{-1}$ | 220 $s^{-1}$ |
| High shear experiments | 1500 $s^{-1}$ | 1100 $s^{-1}$ |

**Table B. Amplitude parameters.**

| | Low shear value | High shear value |
|---|---|---|
| Amplitude parameter ($K_y$) | $1.499600 \times 10^2$ | $4.550000 \times 10^2$ |
| Amplitude parameter ($K_z$) | $1.290096 \times 10^2$ | $3.552400 \times 10^2$ |

# References


[1] Eckstein EC, Belgacem F. Model of platelet transport in flowing blood with drift and diffusion terms. Biophysical journal. 1991;60(1):53–69. doi:10.1016/S0006-3495(91)82030-6.

[2] Leiderman K, Fogelson AL. Grow with the flow: a spatial–temporal model of platelet deposition and blood coagulation under flow. Mathematical medicine and biology: a journal of the IMA. 2011;28(1):47–84. doi:10.1093/imammb/dqq005.
