# Supplementary material for: A three-dimensional shear dependent continuum model of platelet aggregation under flow
Source: PLoS Comput Biol. 2026 May 18;22(5):e1014241. doi: 10.1371/journal.pcbi.1014241 (PMC13218622; doi:10.1371/journal.pcbi.1014241)
Supplement: S5 Appendix — (PDF) [file pcbi.1014241.s005.pdf]

S5 Appendix

Specifications for simulating the mircofluidic channel

The computational domain, Ω is a 3D rectangular channel with width 150 μm, length 150 μm, and height 50 μm. The injury region, $\partial\Omega_{\text{inj}} = [100\ \mu\text{m} \times 100\ \mu\text{m}]$, is centered on the bottom of the computational domain. The timestep for the simulation is 10μs and the reaction sub step factor is 2. The initial 2D simulations to narrow the parameter optimiation window were run on a single Intel Broadwell node with a single CPU core with 256 GB of memory per node. The 3D simulations were run on a single node with 12 cores and 256 GB of memory per node.

Table A. Microfluidic channel boundary conditions.

Boundary	Velocity (U)	Pressure (p)	Biochemical Species	Platelet Species
Inlet	`flowRateInletVelocity`	`zeroGradient`	`zeroGradient`	See S4 Appendix
Outlet	`zeroGradient`	`fixedValue`	`zeroGradient`	`zeroGradient`
Injury wall	`noSlip`	`zeroGradient`	`zeroGradient`	See S1 Appendix
Non-injury wall	`noSlip`	`zeroGradient`	`zeroGradient`	`zeroGradient`

Table B. Microfluidic channel boundary condition parameters.

	Shear rate 300 s^{-1}	Shear rate 1500 s^{-1}
Inlet flow rate (Q_{in})	9.1667×10^{-2} mm^3/s	0.07435×10^{-2} mm^3/s
Kinematic pressure outlet ($\tilde{P}_0$)	0 mm^2/s^2	0 mm^2/s^2

Table C. Microfluidic channel discretization. (I need to confirm that these are indeed the same in the data as these are the tutorial numbers)

Direction	Shear rate 300 s^{-1}	Shear rate 1500 s^{-1}
x	$Nx = 18$; $Nx_{inj} = 50$	$Nx = 18$; $Nx_{inj} = 50$
y	$Ny = 18$	$Ny = 18$
z	$Nz = 87$	$Nz = 87$
max Courant	0.5	0.9

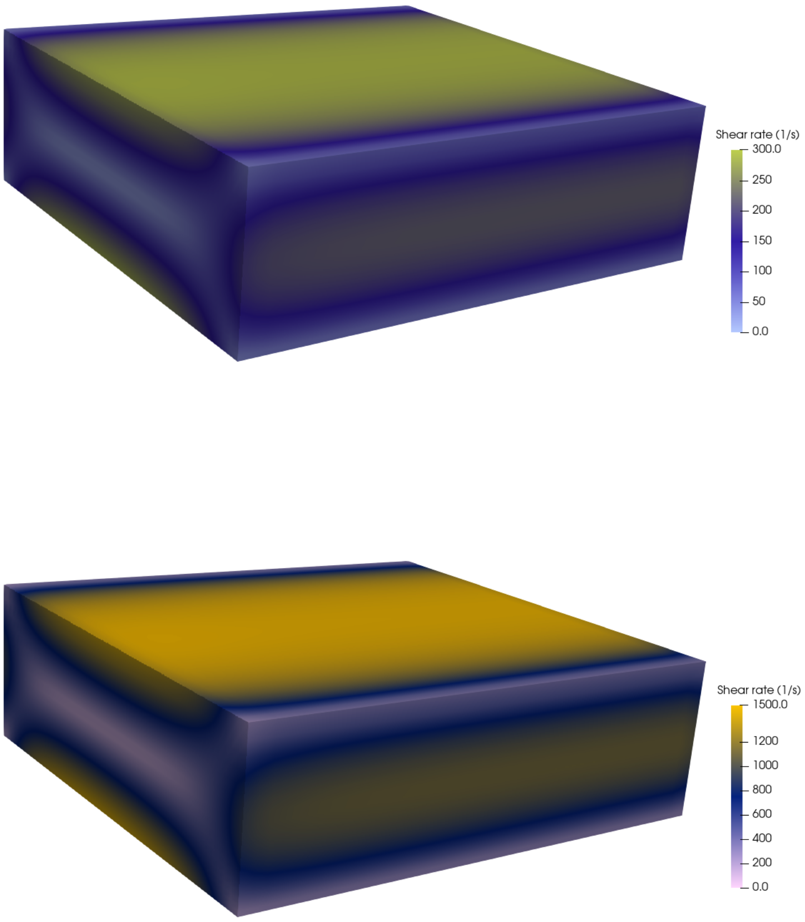

Fig A. Shear rates in computational domain. Top: wall shear-rates for the low shear experiment. Bottom: wall-shear rates for the high shear experiments.
