# Supplementary material for: A three-dimensional shear dependent continuum model of platelet aggregation under flow
Source: PLoS Comput Biol. 2026 May 18;22(5):e1014241. doi: 10.1371/journal.pcbi.1014241 (PMC13218622; doi:10.1371/journal.pcbi.1014241)
Supplement: S6 Appendix — (PDF) [file pcbi.1014241.s006.pdf]

S6 Appendix

Specifications for simulating extravascular injuries

The vertical channels of the microfluidic device are 10 mm long [1], however, the fluid flow is laminar and does not change significantly over the length of the vertical channels. Therefore, we define a computational domain centered around the injury channel that is only 170 μm in length. The mesh is generated using OpenFOAM's `blockMesh` application with vertices and blocks defined as in Fig. A. The open interface where the injury channel meets the vertical channels is created using the `mergePatchPairs` function. The dimensions of the computational are provided in Table A.

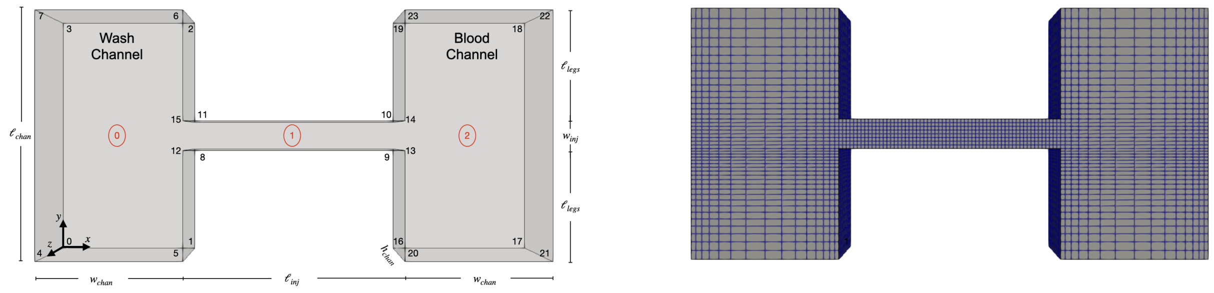

Fig A. Meshing the H-domain. Left: defining the bleeding chip with vertices and blocks for meshing with `blockMesh`. Right: the mesh for the computational domain.

The mesh is defined so that the cells in x and y directions of the injury channel have a uniform spacing of $\Delta x = \Delta y =$2.5 μm. Everywhere else, the mesh is graded for computational efficiency. The resulting mesh that is shown in Fig. A has 28,800 finite volume cells. Simulations of this geometry were performed on an AMD EPYC cluster with utilizing ThinkSystem Mellanox with InfiniBand NDR network. Each node has 1.5TB capacity RAM and 200 cores. Each simulation utilized 48 cores.

Table A. Dimensions of computational domain.

Width of vertical channel (w_{chan})	100 μm
Height of vertical channel (h_{chan})	60 μm
Length of vertical channel (ℓ_{chan})	170 μm
Width of injury channel (w_{inj})	20 μm
Height of injury channel (h_{inj})	60 μm
Length of injury channel (ℓ_{inj})	150 μm

Boundary conditions via hydraulic circuit analysis

In the mircrofluidic device, controllers coupled to flow meters were used to perfuse blood and wash buffer at a flowrate of 5.5 μL/min and 10 μL/min, respectively, through the device [1]. This resulted in an initial pressure drop across the injury channel of $\Delta P_{\text{inj}} = 280.851$ Pa [1, 2]. The outlet pressures for the computational domain are computed using a hydraulic circuit analysis, analogous to Ohms law, for the circuit shown in Fig. B.

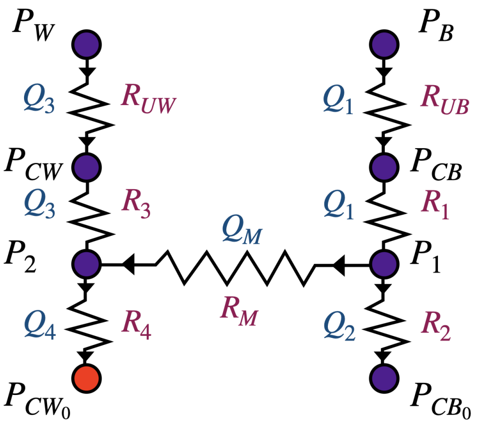

Fig B. The hydraulic circuit. The circuit that determines the required outlet pressure P_{CB_0} given inlet flow rates Q_1 and Q_3, outlet pressure $P_{CB_0} = 0$ Pa, and an injury pressure drop of $\Delta P_{\text{inj}} = 280.851$ Pa.

The pressure gradient between each segment of the circuit ΔP, is proportional to the volumetric flow rate Q. That is, $\Delta P = QR$, where R is the resistance to the flow due to the walls of the channel. The resistance in each channel of the is calculated as [3]:

$$R = \frac{a\mu\ell}{wh^3},$$

$$a = 12\left[1 - \frac{192h}{\pi^5 w}\tanh\left(\frac{\pi w}{2h}\right)\right]^{-1}.$$

We assume that the direction of flow through the injury channel is from the blood to the wash channel. Therefore, the outlet flow rates satisfy:

$$Q_2 = Q_1 - Q_M, \tag{1}$$

$$Q_4 = Q_3 + Q_M. \tag{2}$$

The pressure drops across each segment of the hydraulic circuit are:

$$P_B - P_{CB} = R_{UB}Q_1 \tag{3}$$

$$P_{CB} - P_1 = R_1 Q_1 \tag{4}$$

$$P_1 - P_{CB_0} = R_2 Q_2 \tag{5}$$

$$P_W - P_{CW} = R_{UW}Q_3 \tag{6}$$

$$P_{CW} - P_2 = R_3 Q_3 \tag{7}$$

$$P_2 - P_{CW_0} = R_4 Q_4 \tag{8}$$

$$P_1 - P_2 = R_M Q_M \tag{9}$$

In order to solve this system while ensuring the pressure drop across the injury channel is $\Delta P_{\text{inj}} = 280.851$ Pa, we set $Q_M = \frac{\Delta P_{\text{inj}}}{R_M}$. and $P_{CW_0} = 0$ Pa. The resulting linear system of equations is given by:

$$\begin{bmatrix} 1 & 0 & 0 & 0 & 0 & 0 & 0 & 0 & 0 \\ 0 & 1 & 0 & 0 & 0 & 0 & 0 & 0 & 0 \\ 0 & 0 & 1 & \text{-1} & 0 & 0 & 0 & 0 & 0 \\ 0 & 0 & 0 & 1 & 0 & \text{-1} & 0 & 0 & 0 \\ -R_2 & 0 & 0 & 0 & \text{-1} & 1 & 0 & 0 & 0 \\ 0 & 0 & 0 & 0 & 0 & 0 & 1 & \text{-1} & 0 \\ 0 & 0 & 0 & 0 & 0 & 0 & 0 & 1 & \text{-1} \\ 0 & -R_4 & 0 & 0 & 0 & 0 & 0 & 0 & 1 \\ 0 & 0 & 0 & 0 & 0 & 1 & 0 & 0 & \text{-1} \end{bmatrix} \begin{bmatrix} Q_2 \\ Q_4 \\ P_B \\ P_{CB} \\ P_{CB_0} \\ P_1 \\ P_W \\ P_{CW} \\ P_2 \end{bmatrix} = \begin{bmatrix} Q_1 - Q_M \\ Q_3 + Q_M \\ Q_1 R_{UB} \\ Q_1 R_1 \\ 0 \\ Q_3 R_{UW} \\ Q_3 R_3 \\ P_{CW_0} \\ Q_M R_M \end{bmatrix}. \tag{10}$$

The paramemters used in the hydraulic circuit are:

Table B. Dimensions of hydraulic circuit [2, 1].

Width of vertical channels (w_{chan})	100 μm
Width of injury channel (w_{inj})	20 μm
Length from P_B to P_{CB} (and from P_W to P_{CW})	4915 μm
Length from P_{CB} to P_1 (and from P_{CW} to P_2)	85μm
Length from P_1 to P_{CB_0} (and from P_2 to P_{CW_0})	85 μm
Length of injury channel (ℓ_{inj})	150 μm
Length of injury channel used in HCA ($\ell_{\text{inj,eff}}$)	165μm
Height of vertical channels (h_{chan})	60 μm
Height of injury channel (h_{inj})	60 μm

Table C. Viscocities for HCA [2, 1].

Blood channel viscosity	3.6×10^{-2} Poise
Upstream wash channel viscosity (from P_W to P_2)	1.0×10^{-2} Poise
Injury channel viscosity	2.67×10^{-2} Poise
Downstream wash channel viscosity (from P_2 to P_{CW_0})	2.3×10^{-2} Poise

Table D. Calculated resistances from HCA.

R_{UB}	1.5664×10^{13} Pa s/m^3
R_{UW}	4.3512×10^{12} Pa s/m^3
R_1, R_2	2.7089×10^{11} Pa s/m^3
R_3	7.5249×10^{10} Pa s/m^3
R_4	1.7307×10^{11} Pa s/m^3
R_M	1.2796×10^{13} Pa s/m^3

Table E. Known flow rates for HCA.

Inlet flow rate (Blood, Q_1)	5.5 μL/min
Inlet flow rate (Wash, Q_3)	10 μL/min
Injury flow rate ($Q_M = \Delta P_{\text{inj}}/R_M$)	1.3169 μL/min

Table F. Summary of computed flow rates and pressures from HCA.

Inlet flow rate (Blood, Q_1)	5.5 μL/min
Outlet flow rate (Blood, Q_2)	4.1831 μL/min
Inlet flow rate (Wash, Q_3)	10 μL/min
Outlet flow rate (Wash, Q_4)	11.317 μL/min
Injury flow rate (Q_M)	1.3169 μL/min
Pressure inlet (Blood, P_B)	1.7742×10^3 Pa
Pressure inlet (Wash, P_W)	7.7039×10^2 Pa
Pressure computational inlet (Blood, P_{CB})	3.3833×10^2 Pa
Pressure computational outlet (Blood, P_{CB_0})	2.9461×10^2 Pa
Pressure computational inlet (Wash, P_{CW})	4.5186×10^1 Pa
Pressure computational outlet (Wash, P_{CW_0})	0 Pa
Pressure injury inlet (Wash, P_1)	3.1350×10^2 Pa
Pressure injury outlet (Wash, P_2)	3.2645×10^1 Pa
Pressure drop ($\Delta P_{\text{inj}} = P_1 - P_2$)	2.80851×10^2 Pa

Table G. Boundary conditions for CFD simulation.

Inlet flow rate (Blood, Q_1)	9.1667×10^{-2} mm^3/s	`flowRateInletVelocity`
Inlet flow rate (Wash, Q_3)	1.6667×10^{-1} mm^3/s	`flowRateInletVelocity`
Kinematic pressure outlet (Blood, $\tilde{P}_{CB_0}$)	2.9461×10^5 mm^2/s^2	`fixedValue`
Kinematic pressure outlet (Wash, $\tilde{P}_{CW_0}$)	0 mm^2/s^2	`fixedValue`

Table H. Comparison of HCA with CFD.

	HCA calculation (mm^3/s)	Results from CFD (mm^3/s)
Flowrate inlet (Blood, Q_1)	0.091667	0.091667
Flowrate outlet (Blood, Q_2)	0.069718	0.0713159
Flowrate inlet (Wash, Q_3)	0.16667	1.6667
Flowrate outlet (Wash, Q_4)	0.18862	0.187022
Flowrate injury (Injury, Q_M)	0.021949	0.0191453

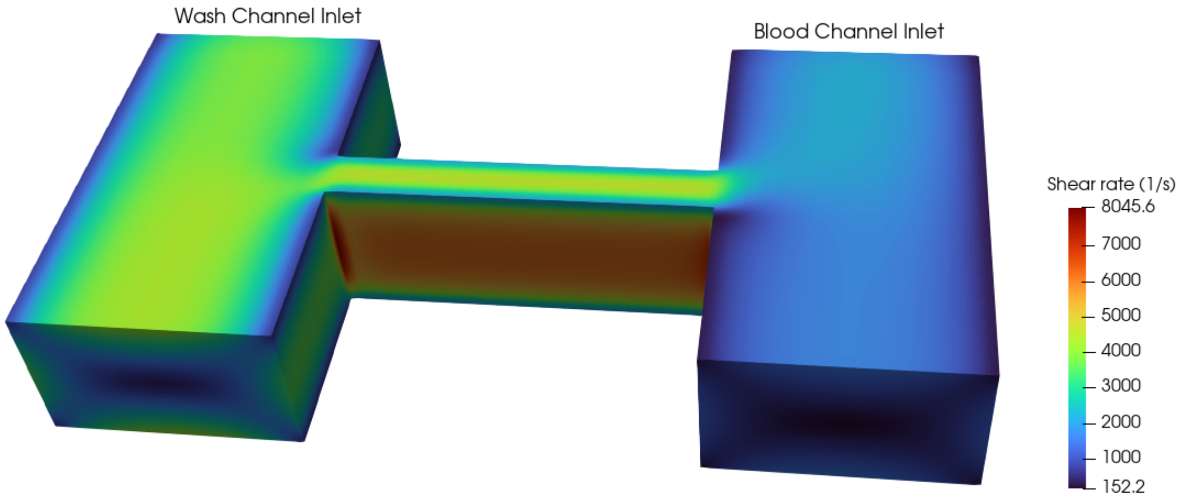

Fig C. Wall shear rates in the extravascular injury domain. The maximum wall shear rate of $\dot{\gamma} = 8045.6\ \text{s}^{-1}$ occurs at the lower interface of the injury channel with the wash channel. The average wall shear rate in the injury channel is $\dot{\gamma} \approx 4410\ \text{s}^{-1}$. In the center of the injury channel, the max wall shear rate on the top (same on bottom) of the channel is $\dot{\gamma} = 5412\ \text{s}^{-1}$, and $\dot{\gamma} = 3855\ \text{s}^{-1}$ on the front (and back) of the channel. These shear rates are within the range of a puncture wound in the saphenous vein of a mouse [4].

References

[1] Link KG, Sorrells MG, Danes NA, Neeves KB, Leiderman K, Fogelson AL. A Mathematical Model OF Platelet Aggregation in an Extravascular Injury Under Flow. Multiscale modeling & simulation. 2020;18(4):1489–1524. doi:10.1137/20M1317785.

[2] Danes NA, Leiderman K. A density-dependent FEM-FCT algorithm with application to modeling platelet aggregation. International journal for numerical methods in biomedical engineering. 2019;35(9):e3212. doi:10.1002/cnm.3212.

[3] Oh KW, Lee K, Ahn B, Furlani EP. Design of pressure-driven microfluidic networks using electric circuit analogy. Lab on a Chip. 2012;12(3):515–545. doi:10.1039/C2LC20799K.

[4] Yakusheva AA, Butov KR, Bykov GA, Závodszky G, Eckly A, Ataullakhanov FI, et al. Traumatic vessel injuries initiating hemostasis generate high shear conditions. Blood Advances. 2022;6(16):4834–4846.
