# Supplementary material for: A three-dimensional shear dependent continuum model of platelet aggregation under flow
Source: PLoS Comput Biol. 2026 May 18;22(5):e1014241. doi: 10.1371/journal.pcbi.1014241 (PMC13218622; doi:10.1371/journal.pcbi.1014241)
Supplement: S7 Appendix — (PDF) [file pcbi.1014241.s007.pdf]

S7 Appendix

Extravascular Clot Structure and Shear-Dependent Morphology

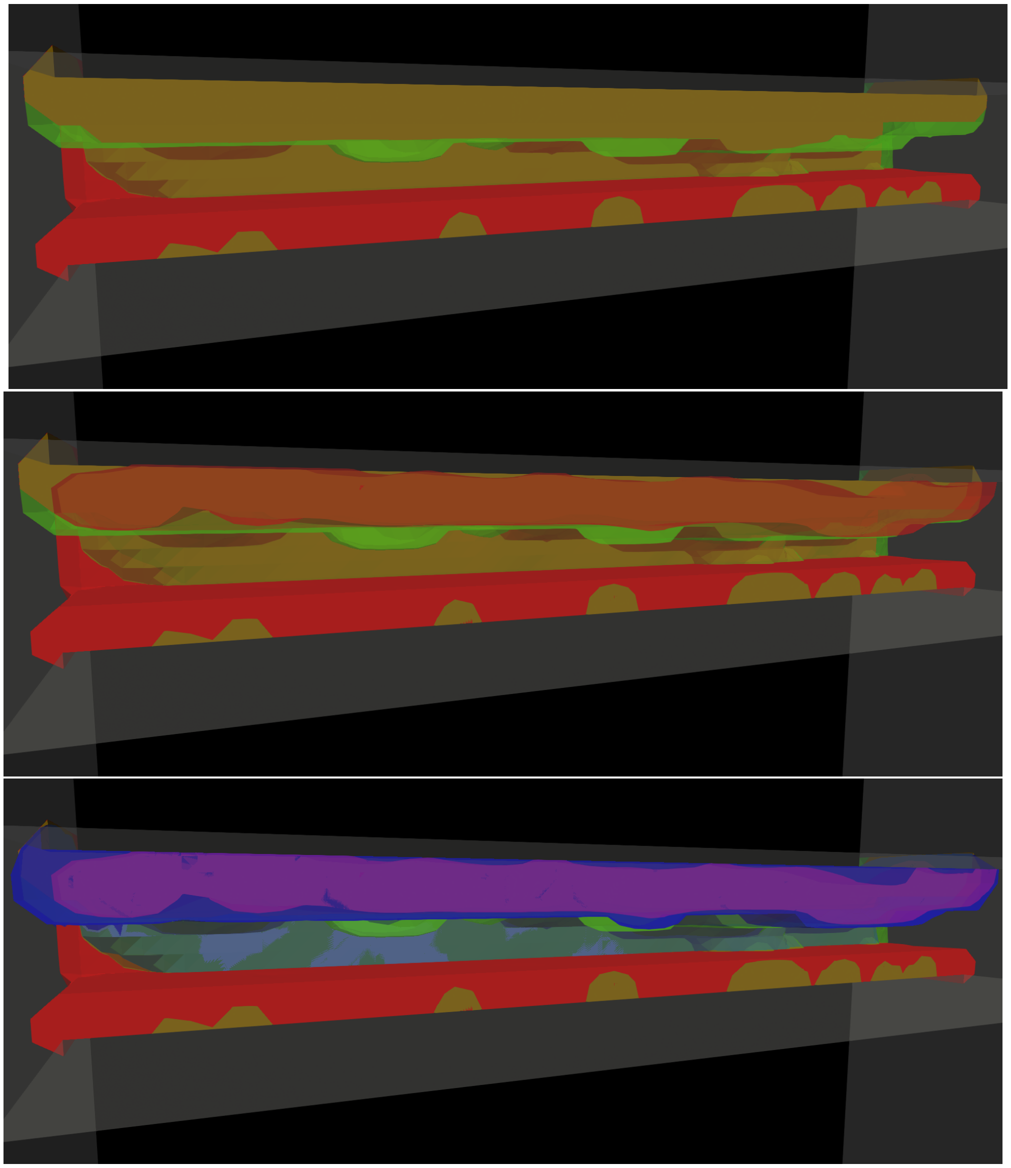

A

B

C

Fig A. 3D visualization of clot structure. Clot structure simulated using model with truncated off rate at 8000/s. (A) Isovolume of subendothelial platelets (red) and bound unactivated platelets (green). (B) Same as in (A), with the addition of activated platelets bound via vWF (red). (C) Same as in (B), with an additional isovolume (blue) representing activated platelets bound via fibrinogen. The isovolumes are sliced along the geometry centerline. Layered together, these renderings illustrate the spatial organization and composition of platelet populations within the clot.

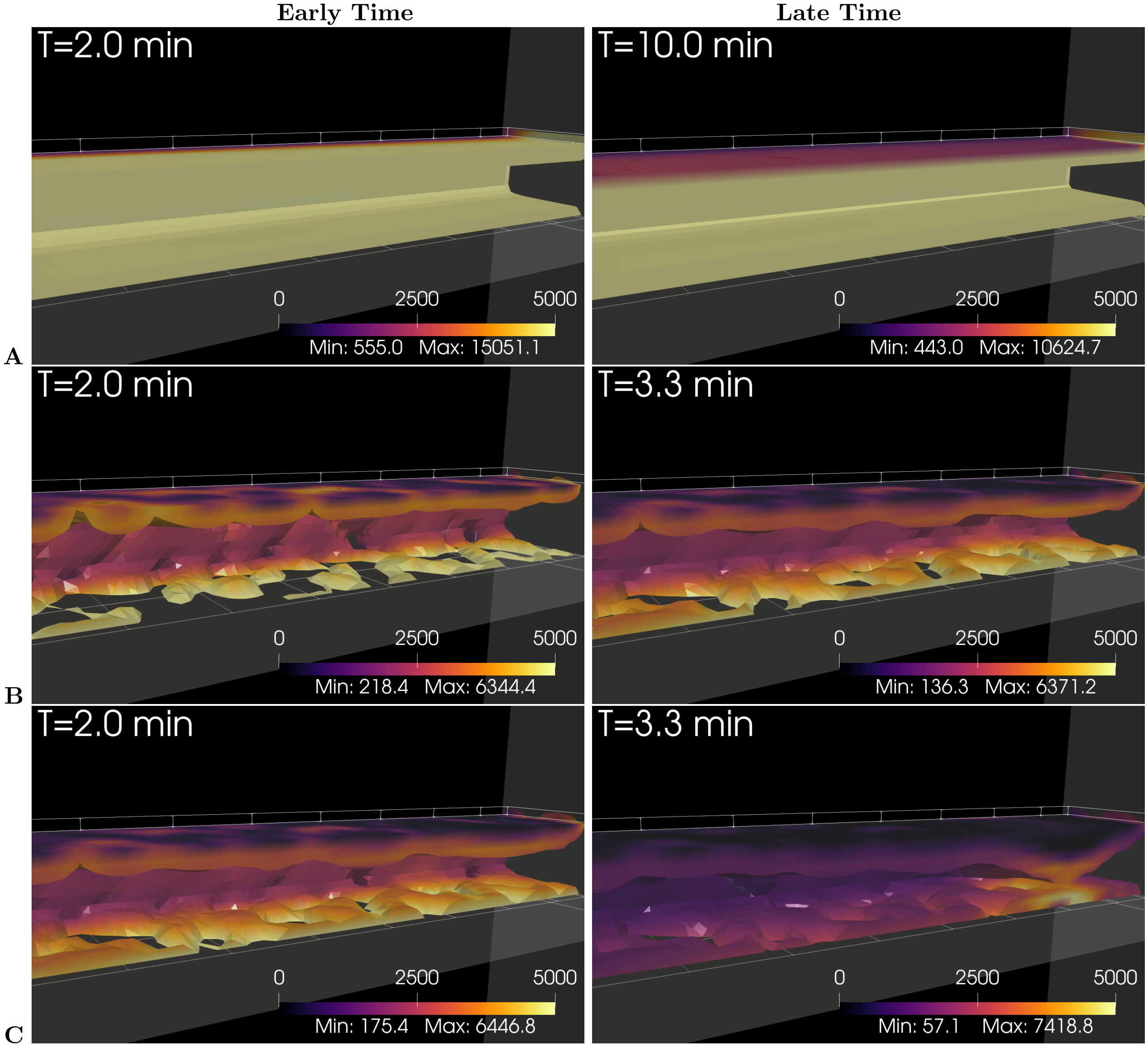

Fig B. 3D visualization of shear-dependent clot morphology. Clot structures at early (left) and late (right) time without shear dependence (A) and with shear-dependent kinetics (B and C). Each clot is shown as an isovolume of bound platelet fraction and sliced longitudinally on the chip centerline. Color contours on the clot surface indicate instantaneous shear rate (1/s). (B) Off-rate truncation at 8000/s. (C) Exponential off-rate increase beginning at 2000/s. Time points are labeled.
