# Supplementary material for: A three-dimensional shear dependent continuum model of platelet aggregation under flow
Source: PLoS Comput Biol. 2026 May 18;22(5):e1014241. doi: 10.1371/journal.pcbi.1014241 (PMC13218622; doi:10.1371/journal.pcbi.1014241)
Supplement: S8 Appendix — (PDF) [file pcbi.1014241.s008.pdf]

S8 Appendix

Donor Blood Information

Table A. Platelet count and hematocrit for each donor. Platelet count is reported in $10^3/\text{mm}^3$.

Medical ID	Shear (s^{-1})	Platelet count	Hematocrit (%)
014	300	219	44.8
015	300	172	46.0
027	300	186	42.9
033	300	201	44.0
035	300	281	37.7
049	300	206	35.2
053	300	319	47.3
058	300	264	42.5
065	300	343	44.3
069	300	227	38.3
074	300	342	40.6
077	300	226	40.7
117	300	266	44.2
124	300	199	40.4
028	1500	200	42.2
065	1500	263	42.1
088	1500	202	42.2
092	1500	339	35.3
101	1500	229	40.7
103	1500	275	48.4
115	1500	310	47.4
127	1500	293	44.2

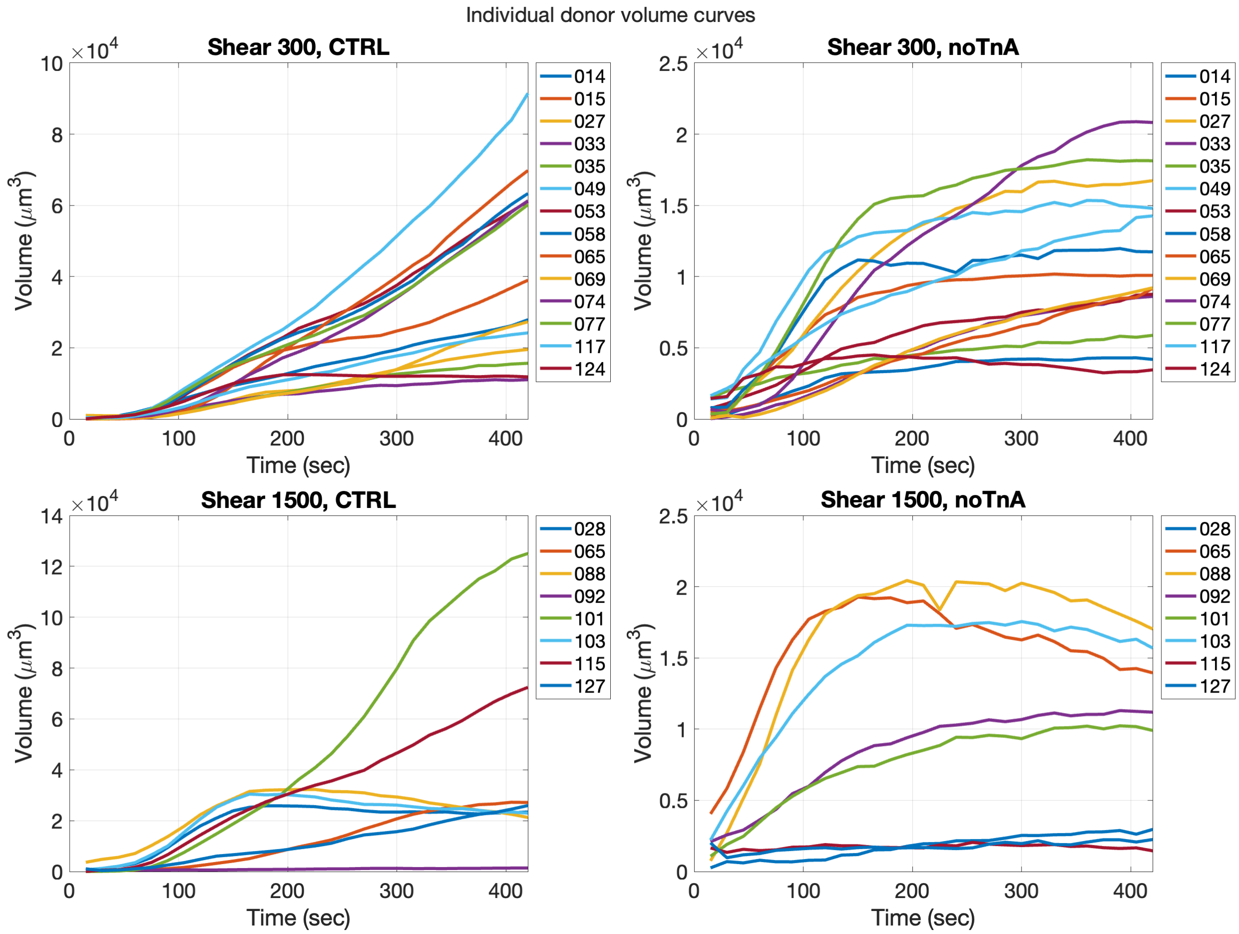

Fig A. Clot volume curves for individual donors.
